# Cell Tropism Predicts Long-term Nucleotide Substitution Rates of Mammalian RNA Viruses

**DOI:** 10.1371/journal.ppat.1003838

**Published:** 2014-01-09

**Authors:** Allison L. Hicks, Siobain Duffy

**Affiliations:** Department of Ecology, Evolution, and Natural Resources, School of Environmental and Biological Sciences, Rutgers, The State University of New Jersey, New Brunswick, New Jersey, United States of America; Universitat de Valencia, Spain

## Abstract

The high rates of RNA virus evolution are generally attributed to replication with error-prone RNA-dependent RNA polymerases. However, these long-term nucleotide substitution rates span three orders of magnitude and do not correlate well with mutation rates or selection pressures. This substitution rate variation may be explained by differences in virus ecology or intrinsic genomic properties. We generated nucleotide substitution rate estimates for mammalian RNA viruses and compiled comparable published rates, yielding a dataset of 118 substitution rates of structural genes from 51 different species, as well as 40 rates of non-structural genes from 28 species. Through ANCOVA analyses, we evaluated the relationships between these rates and four ecological factors: target cell, transmission route, host range, infection duration; and three genomic properties: genome length, genome sense, genome segmentation. Of these seven factors, we found target cells to be the only significant predictors of viral substitution rates, with tropisms for epithelial cells or neurons (*P*<0.0001) as the most significant predictors. Further, one-tailed t-tests showed that viruses primarily infecting epithelial cells evolve significantly faster than neurotropic viruses (*P*<0.0001 and *P*<0.001 for the structural genes and non-structural genes, respectively). These results provide strong evidence that the fastest evolving mammalian RNA viruses infect cells with the highest turnover rates: the highly proliferative epithelial cells. Estimated viral generation times suggest that epithelial-infecting viruses replicate more quickly than viruses with different cell tropisms. Our results indicate that cell tropism is a key factor in viral evolvability.

## Author Summary

RNA viruses are the fastest evolving human pathogens, making their treatment and control difficult. Compared to DNA viruses, RNA viruses replicate with much lower fidelity, which can explain why RNA viruses evolve significantly faster than most DNA viruses. However, there is tremendous variation among the evolutionary rates of different RNA viruses, which is not explained by variation in mutation rates. Here we present a survey of mammalian RNA virus rates of evolution, and a comprehensive comparison of these rates to different properties of virus genomic architecture and ecology. We found that cell tropism is the most significant predictor of long-term rates of mammalian RNA virus evolution. For instance, viruses targeting epithelial cells evolve significantly faster than viruses that target neurons. Our results provide mechanistic insight into why viruses that infect respiratory and gastrointestinal epithelia have been difficult to control.

## Introduction

RNA viruses are responsible for a disproportionate number of emerging human diseases, including influenza, ebola hemorrhagic fever, hantavirus pulmonary syndrome, and Middle East respiratory syndrome, which place tremendous health and economic burdens on both the developing and developed world [1], [2]. In 2008, rotavirus and measles virus caused the deaths of 570,000 children under the age of five, making them two of the leading killers of children worldwide [3]. In 2009, it was estimated that rotavirus infections alone result in $325 million in medical treatment costs and $423 million in societal costs each year [4]. Further, the implementation of many intervention strategies has either failed or been delayed as a result of the evolutionary dynamics of these pathogens [1], [5], [6], [7], [8], [9].

Differences in viral evolutionary dynamics, such as rates of evolution, can explain why certain viruses have the capacity to adapt to new host species, increase in virulence, or develop resistance to antivirals [7], [8], [9], [10], [11]. Therefore, understanding why some RNA viruses evolve more quickly can facilitate better prediction of their pathogenic and epidemiological potential [8], [10], [11], [12]. Though extremely high nucleotide substitution rates are a defining feature of RNA virus evolution [1], [13], [14], [15], there have been few attempts to comprehensively examine the driving genomic and ecological factors behind these rates.

Differences in the strength and direction of selection pressures on these viruses result in variation among their substitution rates [1], [5], [13]. However, while some general patterns have been observed in selection pressures, such as enhanced purifying selection on the structural proteins of arboviruses [16], there have been no attempts to quantify the relationship between selection pressures and long-term viral substitution rates.

The high rates of RNA virus evolution are most commonly attributed to their replication with error-prone RNA-dependent RNA polymerases (RdRps) [1], [17], but these nucleotide substitution rates are known to span at least three orders of magnitude [5], [17] and do not correlate well with experimentally measured viral mutation rates [5]. Further, the substitution rates of some DNA viruses, which replicate with high-fidelity DNA polymerases, are comparable to the high substitution rates of RNA viruses [13]. Therefore, the polymerase error rate alone cannot explain the substitution rate variation in RNA viruses.

Along with mutation rate, viral replication frequency directly impacts the rate at which mutations can be introduced, and ultimately fixed as substitutions [13]. Replication frequencies could be influenced by a variety of factors related to viral genomic architecture or ecology [13]. For example, weak negative correlations between viral genome lengths and substitution rates have been attributed to either enhanced replication frequencies or higher mutation rates in viruses with smaller genomes [15], [17], [18], [19]. It has also been suggested that different transmission and infection modes result in differences in generation time, ultimately causing variation among per-year rates of synonymous substitution of RNA virus structural genes [5].

In this modern survey of mammalian RNA virus evolution rates, we generated and compiled published substitution rates of structural and non-structural genes produced by Bayesian coalescent analyses [20]. We analyzed these rates as a function of seven factors related to virus genomic architecture (*i.e.*, genome length, genome sense, and whether or not the genome is segmented) and virus ecology (*i.e.*, target cell, transmission mode, host range, and whether the infection is acute or persistent). We also evaluated the relationships of viral substitution rates with dN/dS estimates, experimentally measured mutation rates, and estimated generation times. Though recombination undeniably plays a role in shaping viral evolutionary dynamics and could inflate substitution rate estimates [21], [22], we conservatively removed any potential recombinants from our datasets prior to analysis. Through this broad analysis, we were able to demonstrate that cell tropism, and its impact on viral generation time, has the greatest influence on rates of mammalian RNA virus evolution.

## Results

### Datasets

A review of the literature yielded 92 published Bayesian nucleotide substitution rate estimates for the structural genes of 35 different mammalian RNA viral species, and 21 published Bayesian rates for RdRps or a non-structural gene of 14 different viral species (referred to collectively as “non-structural,” Table S1). These rates were supplemented with 26 novel Bayesian substitution rates of structural genes of 19 different viral species, and 19 novel Bayesian rates of non-structural genes of 16 different viral species (Table S2). Collectively, these rates span three orders of magnitude, ranging from 3.0×10^−5^ to 1.5×10^−2^ nucleotide substitutions per site per year (ns/s/y) and 2.0×10^−5^ to 1.3×10^−2^ ns/s/y for the structural genes and non-structural genes, respectively (Table S1).

**Figure 1:**
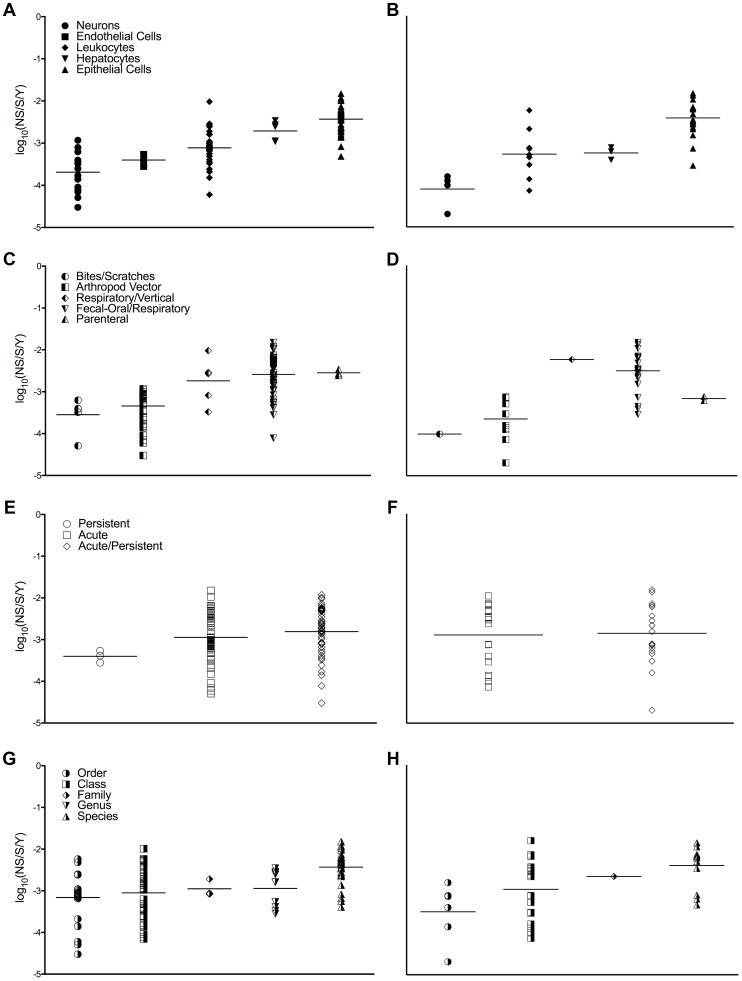
Nucleotide substitution rates and ecological properties of mammalian RNA viruses. Log scale mean substitution rate (log_10_(nucleotide substitutions/site/year, NS/S/Y)) estimates for different target cells (A and B), transmission routes (C and D), infection modes (E and F), and host ranges (G and H). Plots on the left show rates based on structural genes, while the plots on the right show those of non-structural genes. Each black bar indicates the mean of each level, and the levels of each variable are sorted by increasing mean substitution rate. Sources of the rates are given in Table S1.

Plotting the levels of each variable by ascending mean substitution rate revealed similar patterns (*i.e.*, the same ordering of levels) for both the structural (S) and non-structural (NS) datasets in three of these variables, excepting transmission route. Viral substitution rates grouped according to target cell (panels 1A and 1B), transmission route (panels 1C and 1D), infection type (panels 1E and 1F), and host range (panels 1G and 1H) are shown in Figure 1.

**Figure 2:**
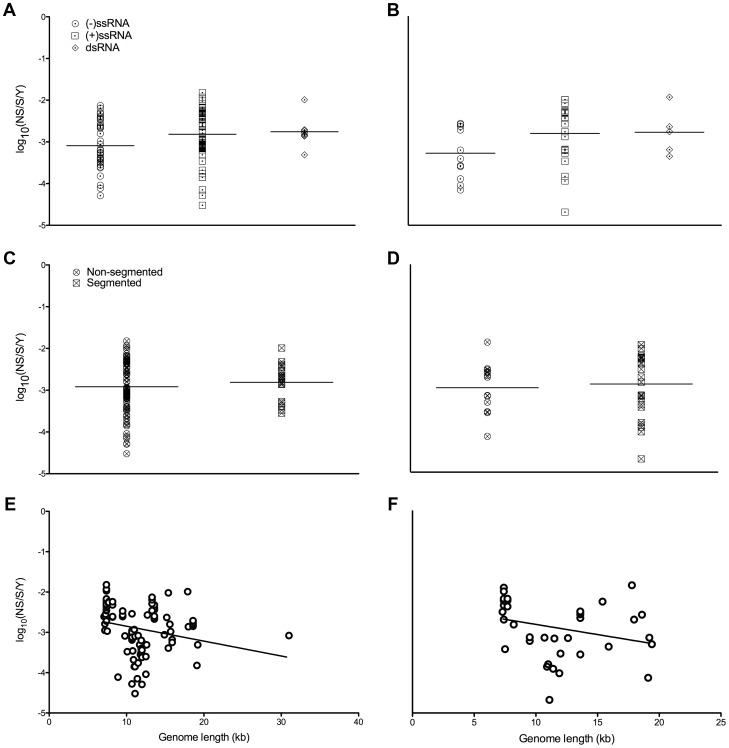
Nucleotide substitution rates and genomic properties of mammalian RNA viruses. Log scale mean substitution rate (log_10_(nucleotide substitutions/site/year, NS/S/Y)) estimates for different genomic architectures (sense/strandedness, A and B, and whether or not the genome is segmented, C and D) and plotted against genome lengths (E and F). The plots on the left show rates based on structural genes, while the plots on the right show those of non-structural genes. Each black bar in A–D indicates the mean of each level, and the levels of each of these variables are sorted by increasing mean substitution rate. The line of best fit is shown in E and F. The coefficients of determination (*R* ^2^) for the linear regression models of genome lengths vs. substitution rates were 0.06 for the structural gene dataset and 0.08 for the non-structural gene dataset. Sources of the rates are given in Table S1.

Substitution rates were also grouped by viral genomic architecture (genome sense/strandedness, Figure 2A and 2B, and genome segmentation, Figure 2C and 2D) and plotted against viral genome length (Figure 2E and 2F). There were no apparent relationships between genomic properties and substitution rates (Figure 2), including no linear relationship between substitution rates and genome lengths in either dataset (coefficient of determination, S: *R* ^2^ = 0.06, NS: *R* ^2^ = 0.08).

dN/dS estimates calculated in this study were compiled with published estimates also calculated using the Single Likelihood Ancestor Counting (SLAC) method (56 structural gene dN/dS estimates, 33 non-structural gene dN/dS estimates total, Table S1).

### Statistical analyses

ANCOVA analyses were performed separately on the structural and non-structural gene datasets to determine which, if any, of seven factors (target cell, transmission route, infection mode, host range, genome length, genome sense, and genome segmentation) significantly predict the nucleotide substitution rates of mammalian RNA viruses. To explore the many dummy-coded categorical variables, three analyses were run using different variable levels as the base levels (see Methods for details, Tables 1 and 2). For all of the ANCOVA analyses, the adjusted coefficient of determination (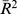) was ≥0.73, indicating that over 70% of the substitution rate variability can be explained by the predictor variables included in this study. Standardized residual plots identified only six potential outliers of the 118 structural gene rates and one potential outlier of the 40 non-structural gene rates (Figure S1), indicating that the data are normally distributed and therefore amenable to a general linear model.

**Table 1:** Significant predictors of viral structural gene substitution rates.

	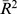	Predictor	β (95% CI)	Significance
1	0.73	Neurons	−0.80 (−1.01, −0.59)	<0.0001
		Leukocytes	−0.56 (−0.80, −0.33)	<0.0001
		Hepatocytes	−0.24 (−0.40, −0.08)	0.0004
		Endothelial cells	−0.18 (−0.31, −0.05)	0.0007
2	0.73	Epithelial cells	1.13 (0.83, 1.43)	<0.0001
		Leukocytes	0.53 (0.33, 0.73)	<0.0001
3	0.73	Neurons	−0.39 (−0.53, −0.24)	<0.0001
		Epithelial cells	0.58 (0.34, 0.82)	<0.0001
	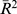	Predictor	β (95% CI)	Significance
1	0.73	Neurons	−0.80 (−1.01, −0.59)	<0.0001
		Leukocytes	−0.56 (−0.80, −0.33)	<0.0001
		Hepatocytes	−0.24 (−0.40, −0.08)	0.0004
		Endothelial cells	−0.18 (−0.31, −0.05)	0.0007
2	0.73	Epithelial cells	1.13 (0.83, 1.43)	<0.0001
		Leukocytes	0.53 (0.33, 0.73)	<0.0001
3	0.73	Neurons	−0.39 (−0.53, −0.24)	<0.0001
		Epithelial cells	0.58 (0.34, 0.82)	<0.0001

For each ANCOVA analysis, the overall adjusted *R* ^2^ (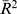) of the model is given along with significant predictor variables (*P*<0.01) and their standardized coefficients (β) with 95% confidence intervals (CIs). In the first ANCOVA, the base levels were epithelial target cells, fecal-oral/respiratory transmission route, acute/persistent infection, species-specific host range, and dsRNA genome architecture. In the second ANCOVA, the base levels were neural target cells, bites/scratches transmission route, persistent infection, order-specific host range, and (−)ssRNA genome architecture. In the third ANCOVA, the base levels were leukocyte target cells, respiratory/vertical transmission route, acute infection, family-specific host range, and (+)ssRNA genome architecture.

**Table 2:** Significant predictors of viral non-structural gene substitution rates.

	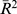	Predictor	β (95% CI)	Significance
1	0.77	-	-	-
2	0.77	-	-	-
3	0.77	Neurons	−0.33 (−1.33, −0.20)	0.009

For each ANCOVA analysis, the overall adjusted *R* ^2^ (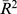) of the model is given along with the significant predictor variable (*P*<0.01) and its standardized coefficients (β) with 95% confidence intervals (CIs). In the first ANCOVA, the base levels were epithelial target cells, fecal-oral/respiratory transmission route, acute/persistent infection, species-specific host range, and dsRNA genome architecture. In the second ANCOVA, the base levels were neural target cells, bites/scratches transmission route, acute infection, order-specific host range, and (−)ssRNA genome architecture. In the third ANCOVA, the base levels were leukocyte target cells, respiratory/vertical transmission route, acute infection, family-specific host range, and (+)ssRNA genome architecture.

**Figure 3:**
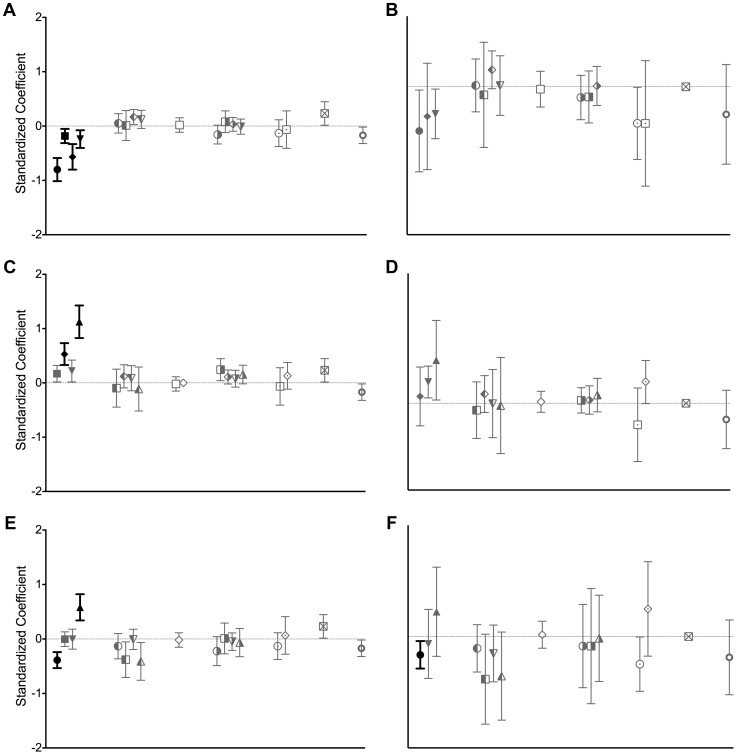
Standardized coefficients for predictors of viral substitution rates. Standardized coefficients with 95% confidence intervals for the different predictor variables of structural (left) and non-structural (right) gene substitution rates. A and B show the coefficients from the first ANCOVA analysis, C and D show coefficients from the second ANCOVA analysis, and E and F show coefficients from the third ANCOVA analysis. Coefficients are indicated by the same symbols used in Figures 1 and 2. Dark coefficients correspond to significant substitution rate predictors (*P*<0.01: neural, leukocyte, hepatocyte, and epithelial target cells in A, leukocyte and epithelial target cells in C, neural and epithelial target cells in E, and neural target cells in F), while the other coefficients are shown in gray.

Regardless of the base levels, target cells were the only significant predictors of log-transformed substitution rates for both structural and non-structural genes (Tables 1 and 2), with cell tropism as the only significant predictor variable by type III sum of squares (SS) analyses (*P*<0.0001 and *P* = 0.003 for the structural and non-structural gene datasets, respectively). Targeting epithelial cells or neurons was found to be the most significant predictor of structural gene rates in each analysis where these were not the base levels (*P*<0.0001, Table 1, Figure 3), while targeting neurons was found to be the sole significant predictor of substitution rates for the smaller non-structural gene dataset (*P* = 0.009, Table 2, Figure 3). Further, there was a high correlation between each viral species' estimated structural gene substitution rate and its corresponding non-structural gene rate (33 viruses, Pearson *r* = 0.87, *P*<0.0001). This suggests that if it were possible to calculate more non-structural rates, we would likely see results similar to those from the structural gene dataset.

To minimize any potential bias introduced by using multiple published rates for a single viral strain or species, we conducted control analyses using datasets with only one rate per species. For species with multiple substitution rates in one of our datasets, we calculated the average log substitution rate and used that as the sole substitution rate for the species in the control analysis. These data were also normally distributed (Figure S2), but the 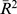 for these analyses were slightly lower than for the full datasets (S: 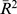 = 0.65, NS: 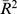 = 0.70, Tables S3 and S4). These control results were consistent with those from the full dataset analyses: tropisms for epithelial cells or neurons were the most significant substitution rate predictors (Tables S3 and S4, Figure S3).

**Figure 4:**
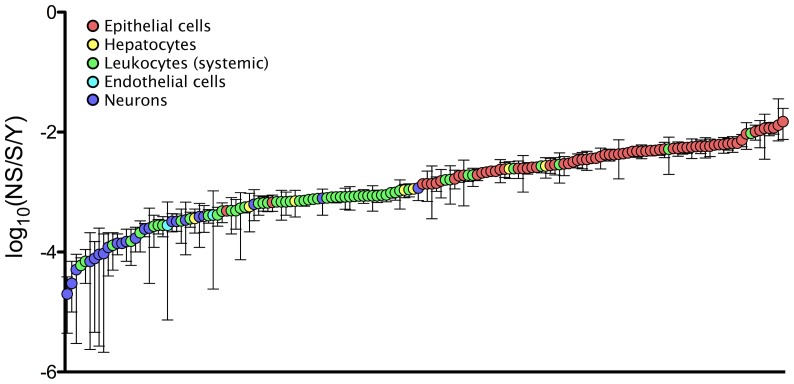
Nucleotide substitution rates and principle target cells of mammalian RNA viruses. Log scale mean nucleotide substitution rates (log_10_(nucleotide substitutions per site per year, NS/S/Y)) of all RNA viruses included in this study with 95% credibility intervals. Credibility intervals that are not visible are eclipsed by the symbol or, in three cases (NoV GII.b, HEV, and TBEV), were not available from the published source. Sources of the rates are given in Table S1.

Because of the high correlation between the structural and non-structural gene rates, we combined the two datasets (Figure 4) and performed a final set of three ANCOVA analyses using this combined dataset. The results from these analyses were nearly identical to those from the structural gene analyses (Table S5). The exception was that, in addition to cell tropism, Type III SS analysis also identified transmission route as a significant predictor variable (*P* = 0.007), though it was still less significant than cell tropism (*P*<0.0001). More specifically, in addition to different cell tropisms, transmission through arthropod vectors was also found to be a significant rate predictor in one of the three analyses (*P* = 0.002, Table S5).

To ensure that any substitution rate variability attributed to a given predictor variable was not significantly dependent on other predictor variables, we examined collinearity in all datasets. With the exception of the persistent infection variable, which was nested with the endothelial target cell variable and thus excluded, the ANCOVA analyses for the structural gene rate datasets and the combined rate dataset showed no significant collinearity (no variance inflation factors (VIF) were greater than 10). For the non-structural gene rate datasets, many different predictor variables had VIF>10. However, subsequent analyses where each individual variable was removed did not significantly reduce collinearity in these datasets (data not shown). Due to the consistent results between the structural and non-structural gene datasets, as well as those from the combined rate dataset, we concluded that correlations among independent variables did not significantly impact our results.

Since target cells were found to be the only consistently significant predictors of substitution rates, a series of one-tailed t-tests was used to confirm which cell tropisms are associated with higher viral substitution rates than others. Viruses that target epithelial cells were found to have significantly higher structural gene substitution rates than viruses that target neurons, endothelial cells, or leukocytes (Table 3, *P*<0.0009). Similarly, viruses that target epithelial cells were found to have significantly higher non-structural gene substitution rates than viruses that target neurons, hepatocytes, or leukocytes (Table 4, *P*<0.0007). These results were recapitulated in the control datasets that only used one rate per viral species (Tables S6 and S7). It should be noted, however, that most of the viruses in this study that are classified as targeting leukocytes ultimately cause systemic infections and infect a wide variety of cell types. Consequently, viruses in the leukocyte target cell category had the most rate variation of all the target cell categories (Figure 1).

**Table 3:** Structural gene substitution rate variation among viruses with different cell tropisms.

	N	En	L	H	Ep
N	-	0.97	1.00	1.00	1.00
En	0.03	-	0.98	1.00	1.00
L	**<0.0001**	0.02	-	0.99	1.00
H	**<0.0001**	0.001	0.006	-	0.98
Ep	**<0.0001**	**0.0008**	**<0.0001**	0.03	-

The significance of viruses with each target cell in the left column having higher log-scale mean substitution rates than the viruses with each target cell in the top row is designated with a p-value from a one-tailed t-test. The threshold for statistical significance (α = 0.01) was Bonferroni-corrected to account for multiple comparisons (*P*<0.001); the significant values are bolded. N = neurons, En = endothelial cells, L = leukocytes, H = hepatocytes, Ep = epithelial cells.

**Table 4:** Non-structural gene substitution rate variation among viruses with different cell tropisms.

	N	L	H	Ep
N	-	0.99	0.99	1.00
L	0.007	-	0.56	1.00
H	0.009	0.44	-	1.00
Ep	**0.0006**	**0.0001**	**0.0001**	-

The significance of viruses with each target cell in the left column having higher log scale mean substitution rates than the viruses with each target cell in the top row is designated with a p-value from a one-tailed t-test. The threshold for statistical significance (α = 0.01) was Bonferroni-corrected to account for multiple comparisons (*P*<0.002); the significant values are bolded. N = neurons, L = leukocytes, H = hepatocytes, Ep = epithelial cells.

**Figure 5:**
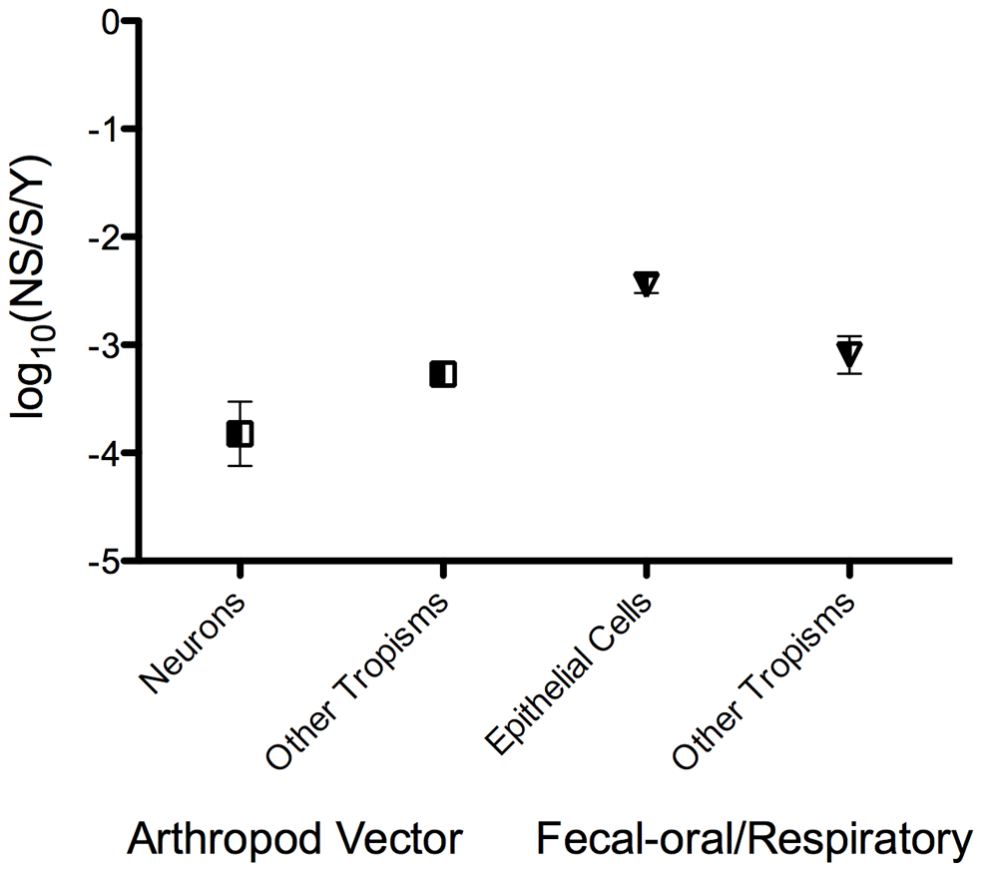
Nucleotide substitution rate variation among arboviruses and fecal-oral/respiratory transmitted viruses with different cell tropisms. The means of the log-scale mean nucleotide substitution rates of neurotopic (n = 13) and non-neurotropic (n = 38) arboviruses are shown as squares on the left, and the means of the log-scale mean nucleotide substitution rates of viruses that are transmitted through the fecal-oral/respiratory routes and primarily target epithelial cells (n = 73) and those that are transmitted through the fecal-oral/respiratory routes and primarily target other cells (n = 15) are shown as the triangles on the right. The mean of each group is shown with 95% confidence intervals (CIs), except for non-neurotropic arboviruses, where the CIs are eclipsed by the symbol.

Because transmission through arthropod vectors was also found to be a significant rate predictor in the ANCOVA analyses based on the combined datasets and because of the correlation between epithelial cell tropism and fecal-oral/respiratory transmission, we evaluated any significant variation among substitution rates of viruses with different transmission routes. Using a series of one-tailed t-tests, we found that viruses that are transmitted through the fecal-oral/respiratory route have significantly higher substitution rates than those transmitted by arthropod vectors (*P*<0.0001). However, we also compared different cell tropisms within each of these transmission routes. We found that fecal-oral/respiratory transmitted viruses that target epithelial cells have significantly higher substitution rates than those that target other cell types (*P*<0.0001, Figure 5). Similarly, we found that neurotropic arboviruses have significantly lower substitution rates than arboviruses that target other cell types (*P*<0.001, Figure 5).

We also tested for linear relationships between viral substitution rates and other evolutionary parameters for which only smaller subsets of our datasets could be analyzed. Reliable experimentally measured mutation rates estimated as mutations per base per infectious cycle were only available for four different viruses included in this study (poliovirus 1 [11], [23], [24], hepatitis C virus [25], influenza A virus [26], [27], [28], influenza B virus [26]). Mutation rates measured as mutations per base per strand replication were only available for three viruses included in this study (poliovirus 1 [29], measles virus [30], [31], and influenza A virus [32]). These mutation rates were not significantly correlated with their corresponding substitution rate estimates (*r* = 0.69, *P* = 0.31 and *r* = −0.93, *P* = 0.25, for mutation rates measured as mutations per base per infection and mutation rates measured as mutations per base per replication, respectively). Similarly, there were no significant correlations between the estimated substitution rates and dN/dS estimates (*ρ* = −0.02, *P* = 0.88 and *ρ* = −0.07, *P* = 0.68, for the limited structural gene and non-structural gene datasets, respectively).

ANCOVA and t-tests consistently revealed epithelial cell tropism and neurotropism as the most significant viral substitution rate predictors. Since these two cell types have some of the highest and lowest turnover rates, respectively, of all mammalian cells [33], [34], [35], [36], we sought to determine if there were any associations between host cell turnover rate and viral generation time. Using the model proposed by Sanjuán (2012) that relates the long-term substitution rate, *K*, to the mutation rate, *μ*, correcting for transient deleterious mutations, we were able to estimate generation times for the few viruses with reliable mutation rate estimates. This model, 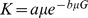, with 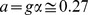, 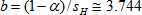 (G = genome length, g = generation time, *s_H_* = harmonic mean of the selection coefficient) [15], confirmed that influenza A virus, influenza B virus, and poliovirus, which target epithelial cells, have substantially shorter generation times (<40 hours) than hepatitis C virus, which targets hepatocytes (>200 hours). These results, while based on a very limited dataset, provide quantitative evidence for a link between cell tropism and generation time. Shorter average generation times lead to more rounds of replication per year, which could neatly explain higher per-year substitution rates.

## Discussion

A variety of intrinsic and ecological factors could plausibly alter the tempo of virus evolution by influencing the rate at which genetic diversity is generated, maintained, and fixed within viral populations. Others have focused on genomic properties as drivers of substitution rate variation [14], [15], [17], [18], demonstrating a weak negative correlation between the genome lengths and substitution rates of RNA viruses [15], [17] or suggesting that ssRNA viruses evolve faster than dsRNA viruses [15]. However, we did not find any significant relationship between genomic properties and substitution rates (Figures 2 and 3). While some have conducted more limited studies on the influence of ecological factors [5], [37], we performed a comprehensive analysis that revealed that cell tropism is a key factor in understanding mammalian RNA viral substitution rates.

It has been proposed that persistent viruses evolve more slowly than those that produce acute infections [1], [5], [15], [38]. Unfortunately, with the exception of latent viruses, which are most commonly retro- or DNA viruses and thus not within the scope in this study, it can be difficult to classify viruses as acute or persistent. The duration of persistence can vary; most persistent viral infections begin with an acute phase and may occasionally be resolved after only this acute phase (*e.g.*, HCV), and many viruses that predominantly result in acute infections occasionally persist [39], [40]. By classifying the viruses in this study as accurately as possible, we found no significant association between infection mode and substitution rate. However, only three viruses in this study, all endothelial-infecting hantaviruses, were classified as strictly persistent. This causes the nesting of the persistent level with tropism for endothelial cells, and the persistent infection variable was therefore excluded from our analyses. Infection duration could be a factor explaining substitution rate variation across the Baltimore classifications of viruses, but there is no evidence that it affects the mammalian RNA virus substitution rates included in this study.

Transmission mode and, less explicitly, host range are frequently invoked as determinants of viral substitution rates [5], [41]. Specifically, plant or animal viruses that primarily rely on arthropod vectors for transmission, and therefore obligately infect very diverse hosts, are thought to evolve more slowly than viruses with other transmission modes [5], [41], [42], [43]. Surprisingly, only one of our 15 ANCOVA analyses implicated transmission route as a significant substitution rate predictor, and we found no significant relationship between substitution rate and host range.

The seven genomic and ecological factors examined are not necessarily independent. For example, 25% of the arboviruses in our study are neurotropic, the second-most common cell tropism of our arboviruses (Table S1). Therefore, the observation that vector-borne viruses tend to evolve more slowly is qualitatively consistent with our results. Cell tropism does appear to be the more significant factor, though, as our results show that arboviruses with other cell tropisms evolve significantly faster than those with neurotropism. Previous studies have also indicated that phylogenetic relationships are predictive – that sister taxa have similar rates of evolution [5]. We initially included virus families as an explanatory variable in our analyses, but we had to discard it due to high colinearity with these other seven variables (data not shown). Once the virus families were removed, there was no statistically significant colinearity within the structural gene dataset. Of these seven non-colinear factors, cell tropism was the best predictor of viral substitution rates. The smaller non-structural gene dataset, on the other hand, had significant collinearity among predictor variables that could not be resolved. The NS dataset also had only 1/3 of the taxa, inherently reducing its statistical power. It was not possible to expand the mammalian RNA virus NS dataset at this time; our novel rate analyses increased the number of reliable rates by 40% by exhaustively searching the available sequences in GenBank. The results of the combined dataset were nearly identical to those from the dataset of only S rates, again identifying target cells as the only consistent predictor variables. While many factors likely influence nucleotide substitution rates, and there may be inherent relationships among some of our seven variables, our results affirm that cell tropism is the most significant predictor of mammalian RNA virus substitution rate.

Though previously unexplored, cell tropism could influence viral substitution rates by the same mechanisms that have been suggested for the other ecological factors described above [44]. Infection of different host cells could expose viruses to different selection pressures, which could influence the rates at which mutations are fixed as substitutions. Additionally, it is possible that cell tropism influences the rate at which genetic diversity is generated by affecting viral mutation rates or generation times.

### Selection pressures do not predict substitution rates

Variation in strength and/or direction of selection has frequently been invoked as a determinant of viral substitution rates [12], [13], [21]. While positive selection can certainly result in variation among very short-term substitution rates, purifying selection tends to dominate over longer timescales [21], [45], [46], [47]. However, variation is observed in the strength of purifying selection due to differences in host ranges. For instance, as previously mentioned, viruses vectored by arthropods have unique evolutionary constraints placed on them by their host diversity [41], [42], [43], [48]. While previous studies found that arboviruses are under stronger purifying selection than non-arboviruses [1], [41], [49], we found that the dN/dS estimates based on structural genes of arboviruses were not significantly lower than those for non-arboviruses (*P* = 0.19). The dN/dS estimates based on non-structural genes of arboviruses were only moderately lower than those for non-arboviruses (*P* = 0.04). Further, we found no significant correlation between the estimated dN/dS and substitution rates, suggesting that detectable differences in selection pressures do not explain the variation in substitution rates of mammalian RNA viruses. To date, there are no data supporting a link between cell tropism and sustained differences in selection pressures.

### Mutation and substitution rates are uncorrelated

Compared to the slower evolution of DNA viruses, the evolution of RNA viruses is dominated by their high mutation rates [1], [13], [15]. Weak negative correlations between genome lengths and viral substitution rates have been attributed to a relationship between mutation rate and substitution rate, as smaller genomes could in theory withstand higher mutation rates than larger genomes [13], [15], [50]. However, while differences in spontaneous mutation rates appear to be significantly correlated to the long-term substitution rates of DNA viruses [15], this linear relationship disappears past a certain mutation rate threshold: around 10^−6^ mutations per site per infectious cycle, the lower end of the mutation rate range of RNA viruses [13], [15]. It is, therefore, not surprising that we found no significant correlation between substitution rates and the available, reliable mutation rate estimates. Additionally, a recent study of the retrovirus HIV-1 found that infection of different cell types did not lead to differences in mutation rate [51], providing some evidence that mutation rate is not correlated with cell tropism. Together, these data suggest that mutation rate variation among different cell types is not driving higher substitution rates in epithelial-infecting mammalian RNA viruses.

### Generation time could explain substitution rate variation

Ruling out selection, mutation rates, and recombination frequencies as drivers of RNA virus substitution rates implies that the rate variation is largely the result of variation in replication dynamics [5], [13]. Enhanced replication frequencies (shorter generation times) have been used to explain a variety of the previously suggested links between virus ecology and substitution rate. For example, viruses in the acute phase of an infection generally replicate more frequently than those in a persistent infection, and viruses in a latent phase do not replicate at all [39]. Further, as an alternative to differential selection pressures, the argument that transmission mode drives viral substitution rates assumes that viruses that can be transmitted more rapidly will have shorter generation times (*e.g.*, horizontal transmission vs. vertical transmission [5], [52], [53]).

DNA viruses have shorter generation times in faster dividing cells [54], [55], but the associations between cell tropism and RNA virus generation time are less obvious, as RNA viruses do not depend on cellular replication machinery. However, there is evidence that for at least some RNA viruses, viral genome replication is highly dependent on host cell proliferation, with RNA synthesis occurring at much lower rates in poorly proliferating cells than in rapidly dividing cells [56], [57], [58], [59], [60]. For example, it has been repeatedly demonstrated that hepatitis C virus genome replication is enhanced in proliferating cells, perhaps due to higher levels of available nucleotides [59], or because of higher levels of viral protein synthesis facilitated by nuclear translation initiation factors that only become available in the cytoplasm during cell division [58]. Similar dependence on cell proliferation for viral replication efficiency has been demonstrated in a number of picornaviruses [57], [60], [61], [62]. Further, using the model proposed by Sanjuán (2012), we found that viruses that infect epithelial cells have generation times that may be as much as 40-fold shorter than a virus that infects non-epithelial cells. This offers a possible mechanistic basis for our finding that viruses that target the fastest-dividing cells in the body (intestinal and respiratory epithelial cells [34], [35], [36], [63]) have higher substitution rates than viruses that infect cells that turnover at very low rates, if at all (neurons [33], [35], [64]).

We are the first to provide statistical evidence that cell tropism predicts rates of mammalian RNA virus evolution, likely through its influence on virus generation time. These results offer a new perspective on why it has been difficult to create effective vaccines for viruses that infect epithelial tissue, such as rotavirus and enterovirus 71 [65], [66]. Further, as it has been shown that higher rates of viral evolution can result in increased genetic diversity and higher epidemiological fitness [26], [67], [68], the higher substitution rates of epithelial-infecting viruses predict increased evolvability and greater potential for emergence in novel host species [21].

## Materials and Methods

### Published rates

Long-term nucleotide substitution rates of mammalian RNA viruses were collected from the literature, with a focus on finding rates for the outer structural gene containing the major antigenic site(s) and non-structural (preferably the RdRp) genes. While the RdRp genes of the (-)ssRNA and dsRNA viruses are classified as structural, or virion-associated, genes [69], they are generally thought to be more conserved and under very different selection pressures than the structural genes that interact with the host immune system [70], [71]. We excluded retroviruses from analysis because they are known to have highly variable substitution rates due to time spent integrated into DNA genomes, where they evolve at the rate of their hosts' genome [13], [72]. Viruses that predominately infect non-mammals, with mammals serving as incidental, dead-end hosts, were also excluded. Only rates estimated for individual viral species or strains were used, not those that aggregated multiple species into one analysis. Similarly, only rates from single gene analyses were included, not those based on full genomes or multiple gene alignments. In order to minimize any rate discrepancies that could result from variations among datasets (*e.g.*, number of taxa, temporal range, portion of gene analyzed) and/or subtle methodological variations [45], [73], [74], [75], [76], [77], only rates produced by Bayesian coalescent analyses of datasets composed of at least 30 taxa, isolated over a minimum range of 15 years and spanning at least 40% of the analyzed gene were included. Bayesian coalescent analyses provide estimates of viral evolution that are calculated over a longer range than simply the date range over which the taxa were isolated. This is because they determine the likely phylogenetic relationship among the isolates and infer substitution rates over the entire evolutionary history of the sampled taxa: over decades, hundreds, even thousands of years. These rates can therefore be considered “long-term” nucleotide substitution rates.

Data regarding genomic architecture and ecology were obtained for all viruses with published substitution rates that met these criteria. We included multiple rates for a given virus when available, except when a single study examined multiple lineages and summarized the results in a single rate [78], [79], [80], [81]. Corresponding dN/dS estimates were collected when available.

### Sequence data

These published substitution rates were supplemented with novel BEAST [20] rate analyses based on the sequence data available in GenBank (accessed through Taxonomy Browser, http://www.ncbi.nlm.nih.gov/Taxonomy). Sequences for structural and non-structural genes with years of isolation available in GenBank or the literature were manually aligned using Se-Al v2.0a11 [82]. Sequences with GenBank or published information that indicated they were genetically manipulated or extensively passaged in the lab prior to sequencing were eliminated from further analysis. The final datasets also adhered to the conservative criteria described above for published datasets.

### Substitution rate and selection analyses

As recombination events can lead to over-estimation of nucleotide substitution rates, each dataset was scanned for recombination using seven different algorithms (RDP, GENECONV, Bootscan, MaxChi, Chimaera, SiScan, and 3seq) implemented in RDP v3.44 [83]. Sequences implicated as recombinant by two or more algorithms were excluded from further analysis. These finalized alignments were deposited into Dryad (doi:10.5061/dryad.58ss8). Modeltest v3.7 [84] was used to determine the best-fit model of nucleotide substitution for each dataset (by AIC).

Long-term nucleotide substitution rates were estimated using BEAST v1.5.4 [20]. Each dataset was run for at least 50 million generations and until all parameters had stabilized (effective sampling size >200). Each dataset was run with two different clock models (strict and uncorrelated lognormal) and three different demographic models (constant, exponential, and Bayesian skyline). The best-fitting clock/demographic model combination for each dataset was determined using Bayes factors as implemented in Tracer v1.5 [85]. For each best set of priors, two independent runs were performed to ensure that the results were replicable, and a control analysis was run without the dataset to ensure that the priors were not controlling the outcome of the analysis.

The Single Likelihood Ancestor Counting (SLAC), codon-based maximum likelihood method available in the HYPHY package on the Datamonkey web server [86] was used to evaluate the strength of selection pressure on these datasets.

### Statistical analyses

In order to determine which factors most significantly predict substitution rates of mammalian RNA viruses, ANCOVA analyses were run using SPSS Statistics v21 (IBM) with log-transformed mean substitution rates as the dependent variable and seven overarching predictor variables (target cell, transmission route, whether the infection is acute or persistent, host range, genome length, genome sense, and whether or not the genome is segmented). For each variable, different base levels were tested to ensure that the chosen base level did not significantly influence the results. Collinearity among the variables was also assessed, with variance inflation factors (VIF) greater than 10 indicating redundancy among variables. Separate ANCOVA analyses were run on the structural and non-structural gene datasets. As there were multiple published rates for some viral species and strains, additional analyses were run for both the S and NS datasets with only one substitution rate per virus species. When there were multiple rates for a given virus species, we calculated and used an average rate.

One-tailed t-tests were subsequently run in R v2.14.1 [87] to provide an additional measure of significant directional variation among the log-transformed mean rates of different levels for any categorical variable that was found to be a significant rate predictor (α = 0.01, adjusted by Bonferroni correction for multiple comparisons) in the ANCOVA analyses. Additional t-tests were also conducted using the control datasets with one rate per virus species.

Additionally, though there were no dN/dS or mutation rate estimates available for all viruses used in this study, the available data for each variable were compared to corresponding log-transformed mean substitution rate estimates using Spearman rank correlation (for dN/dS) or Pearson correlation coefficient (for mutation rates). Structural and non-structural gene rate estimates were also compared using Pearson correlation coefficient. All correlation analyses were performed in SPSS Statistics v21.

## Supporting Information

Figure S1 **Standardized residuals of the ANCOVA analyses.** Standardized residuals are shown for each data point, or observation, included in the ANCOVA analyses. A and B show the residuals from the first analysis, C and D show residuals from the second analysis, and E and F show residuals from the third analysis. Residuals outside the interval [−1.96, 1.96] are shown in red and labeled according to the virus abbreviations given in Table S1.
(TIFF)

Figure S2 **Standardized residuals of the ANCOVA analyses using the control datasets.** Standardized residuals are shown for each data point, or observation, included in the ANCOVA analyses using the datasets with one rate per viral species. A and B show the residuals from the first analysis, C and D show residuals from the second analysis, and E and F show residuals from the third analysis. The one residual outside the interval [−1.96, 1.96] is shown in red and labeled according to the virus abbreviations given in Table S1.
(TIFF)

Figure S3 **Standardized coefficients for predictors of viral substitution rates based on analyses of control datasets.** Standardized coefficients with 95% confidence intervals for the different predictor variables of structural (left) and non-structural (right) gene substitution rates, using the datasets with one rate per viral species. A and B show the coefficients from the first analysis, C and D show coefficients from the second analysis, and E and F show coefficients from the third analysis. Coefficients are indicated by the same symbols used in Figures 1 and 2. Dark coefficients correspond to significant substitution rate predictors (*P*<0.01, epithelial, leukocyte, hepatocyte, and epithelial target cells in A, leukocyte and epithelial target cells in C, neural and epithelial target cells in E, and neural target cells in F), while the other coefficients are shown in gray.
(TIFF)

Table S1 **Nucleotide substitution rates and characteristics of all viruses used in this study.**
(DOCX)

Table S2 **Dataset and analysis information for novel substitution rates produced in this study.** Abbreviations for viruses and genes are as in Table S1. Nucleotide substitution models shown general time reversible (GTR), Tamura-Nei (TrN), transition (TIM), transversion (TVM), transversion with equal frequencies (TVMef), Kimura 3-parameter with unequal frequencies (K81uf), and Hasegawa-Kishino-Yano (HKY); corrections for invariant sites (+i) and a gamma distribution of rate heterogeneity (+G) were also included in some models.
(DOCX)

Table S3
**Significant predictors of viral structural gene substitution rates using one rate per viral species.** For each multiple regression analysis, the overall adjusted *R* ^2^ (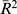) of the model is given along with significant predictor variables (*P*<0.01) and their standardized coefficients (β) with 95% confidence intervals (CIs). In the first regression, the base levels were epithelial target cells, fecal-oral/respiratory transmission route, acute/persistent infection, species-specific host range, and dsRNA genome architecture. In the second regression, the base levels were neural target cells, bites/scratches transmission route, persistent infection, order-specific host range, and (−)ssRNA genome architecture. In the third regression, the base levels were leukocyte target cells, respiratory/vertical transmission route, acute infection, family-specific host range, and (+)ssRNA genome architecture.(DOCX)

Table S4
**Significant predictors of viral non-structural gene substitution rates using one rate per viral species.** For each multiple regression analysis, the overall adjusted *R* ^2^ (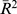) of the model is given along with significant predictor variables (*P*<0.01) and their standardized coefficients (β) with 95% confidence intervals (CIs). In the first regression, the base levels were epithelial target cells, fecal-oral/respiratory transmission route, acute/persistent infection, species-specific host range, and dsRNA genome architecture. No factors were significant in this analysis. In the second regression, the base levels were neural target cells, bites/scratches transmission route, acute infection, order-specific host range, and (−)ssRNA genome architecture. No factors were significant in this analysis. In the third regression, the base levels were leukocyte target cells, respiratory/vertical transmission route, acute infection, family-specific host range, and (+)ssRNA genome architecture.(DOCX)

Table S5
**Significant predictors of viral substitution rates based on all rates included in this study.** For each ANCOVA analysis, the overall adjusted *R* ^2^ (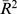) of the model is given along with the significant predictor variable (*P*<0.01) and its standardized coefficients (β) with 95% confidence intervals (CIs). In the first ANCOVA, the base levels were epithelial target cells, fecal-oral/respiratory transmission route, acute/persistent infection, species-specific host range, and dsRNA genome architecture. In the second ANCOVA, the base levels were neural target cells, bites/scratches transmission route, acute infection, order-specific host range, and (−)ssRNA genome architecture. In the third ANCOVA, the base levels were leukocyte target cells, respiratory/vertical transmission route, acute infection, family-specific host range, and (+)ssRNA genome architecture.(DOCX)

Table S6 **Structural gene substitution rate variation among viruses with different cell tropisms.** Based on the control datasets with one substitution rate per viral species. The significance of viruses with each target cell in the left column having higher log scale mean substitution rates than the viruses with each target cell in the top row is designated with a p-value from a one-tailed t-test. The threshold for statistical significance (*P*<0.01) was Bonferroni-corrected to account for multiple comparisons (*P* = 1×10^−3^). N = neurons, En = endothelial cells, L = leukocytes, H = hepatocytes, Ep = epithelial cells.
(DOCX)

Table S7 **Non-structural gene substitution rate variation among viruses with different cell tropisms.** Based on the control datasets with one substitution rate per viral species. The significance of viruses with each target cell in the left column having higher log scale mean substitution rates than the viruses with each target cell in the top row is designated with a p-value from a one-tailed t-test. The threshold for statistical significance (*P*<0.01) was Bonferroni-corrected to account for multiple comparisons (*P*<2×10^−3^). N = neurons, L = leukocytes, H = hepatocytes, Ep = epithelial cells.
(DOCX)
