# Supplementary figures and images for: Cell Tropism Predicts Long-term Nucleotide Substitution Rates of Mammalian RNA Viruses
Source: PLoS Pathog. 2014 Jan 9;10(1):e1003838. doi: 10.1371/journal.ppat.1003838 (PMC3887100; doi:10.1371/journal.ppat.1003838)

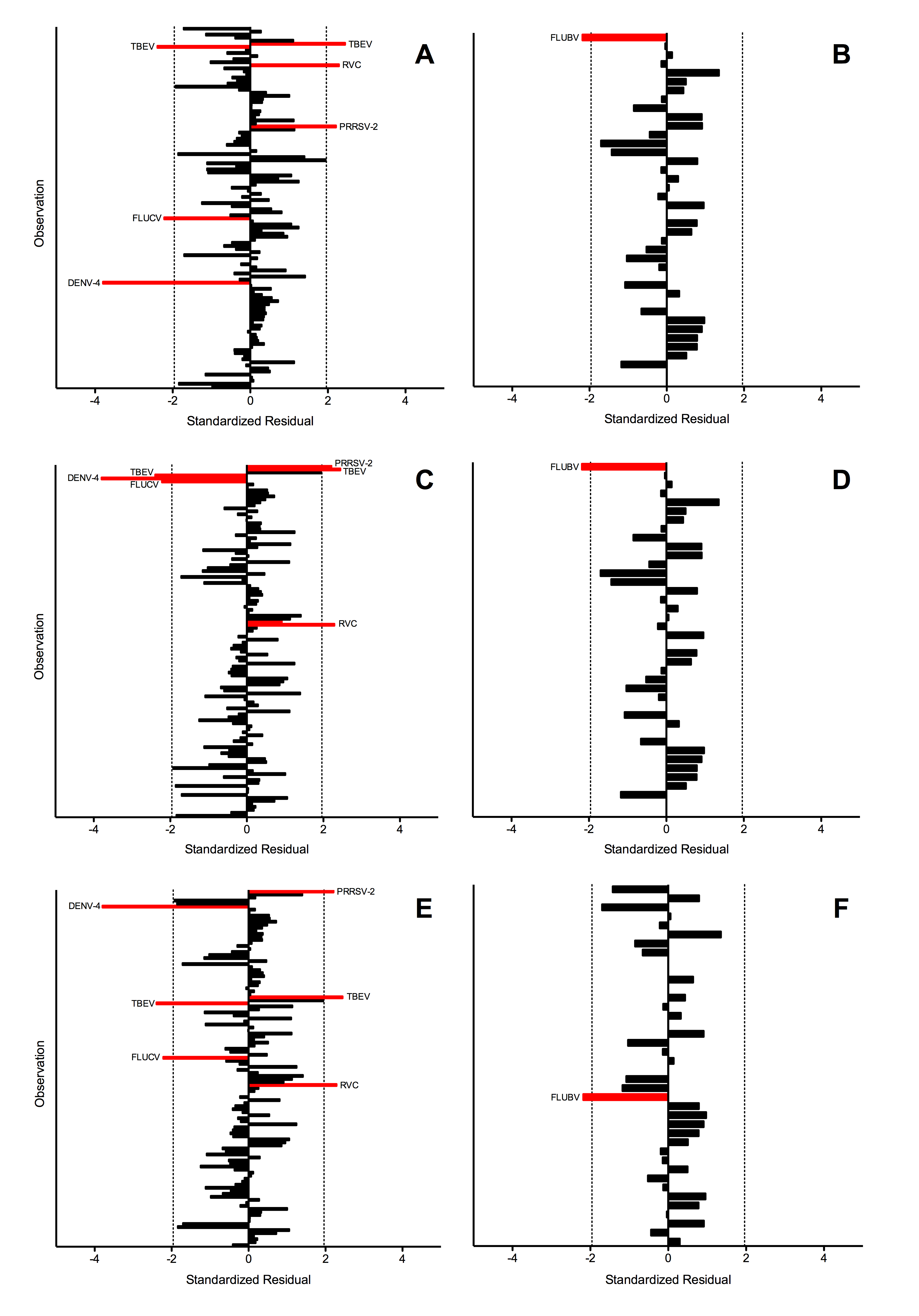

Supplement: Figure S1 — Standardized residuals of the ANCOVA analyses. Standardized residuals are shown for each data point, or observation, included in the ANCOVA analyses. A and B show the residuals from the first analysis, C and D show residuals from the second analysis, and E and F show residuals from the third analysis. Residuals outside the interval [−1.96, 1.96] are shown in red and labeled according to the virus abbreviations given in Table S1. (TIFF) [file ppat.1003838.s001.tiff]

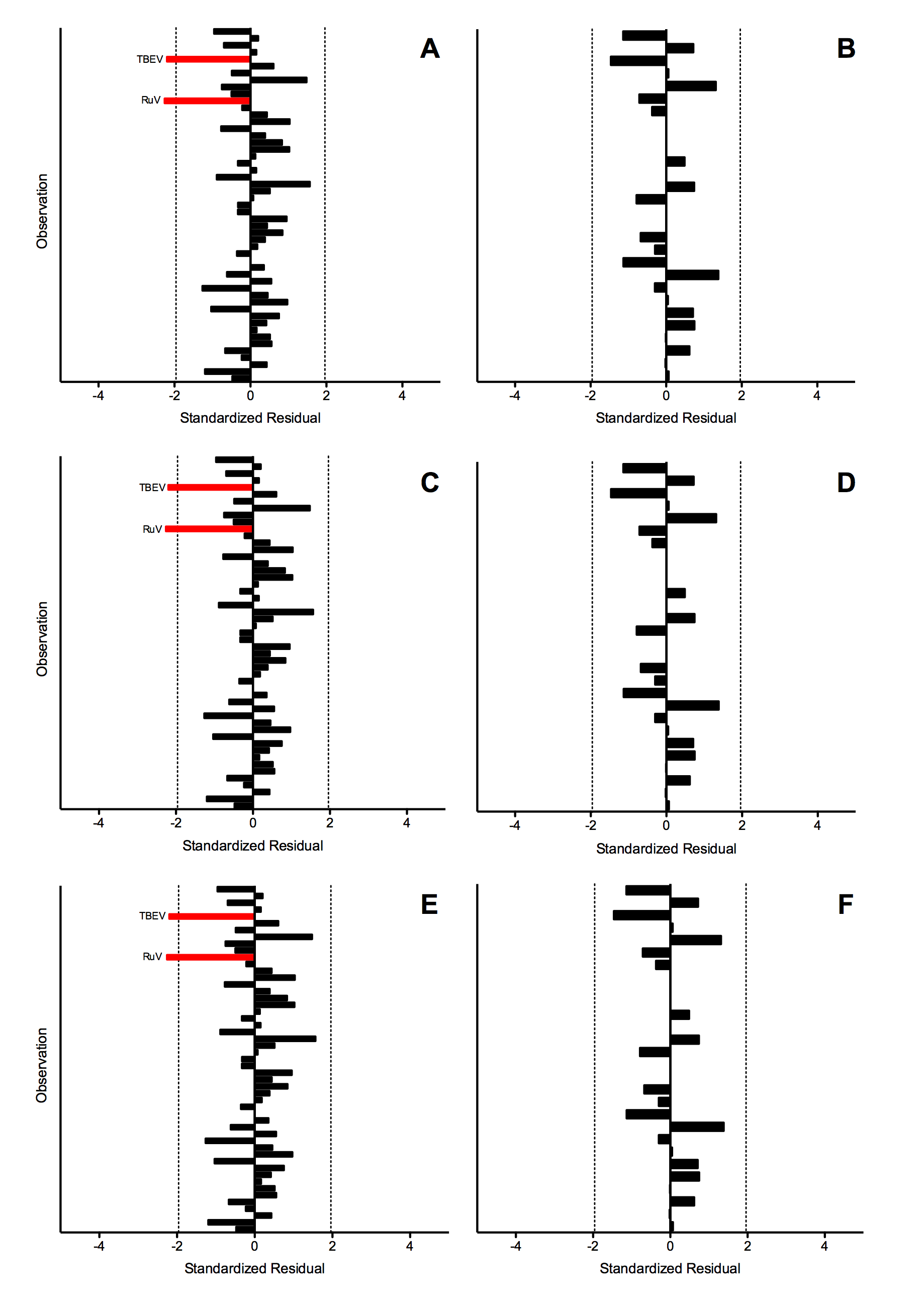

Supplement: Figure S2 — Standardized residuals of the ANCOVA analyses using the control datasets. Standardized residuals are shown for each data point, or observation, included in the ANCOVA analyses using the datasets with one rate per viral species. A and B show the residuals from the first analysis, C and D show residuals from the second analysis, and E and F show residuals from the third analysis. The one residual outside the interval [−1.96, 1.96] is shown in red and labeled according to the virus abbreviations given in Table S1. (TIFF) [file ppat.1003838.s002.tiff]

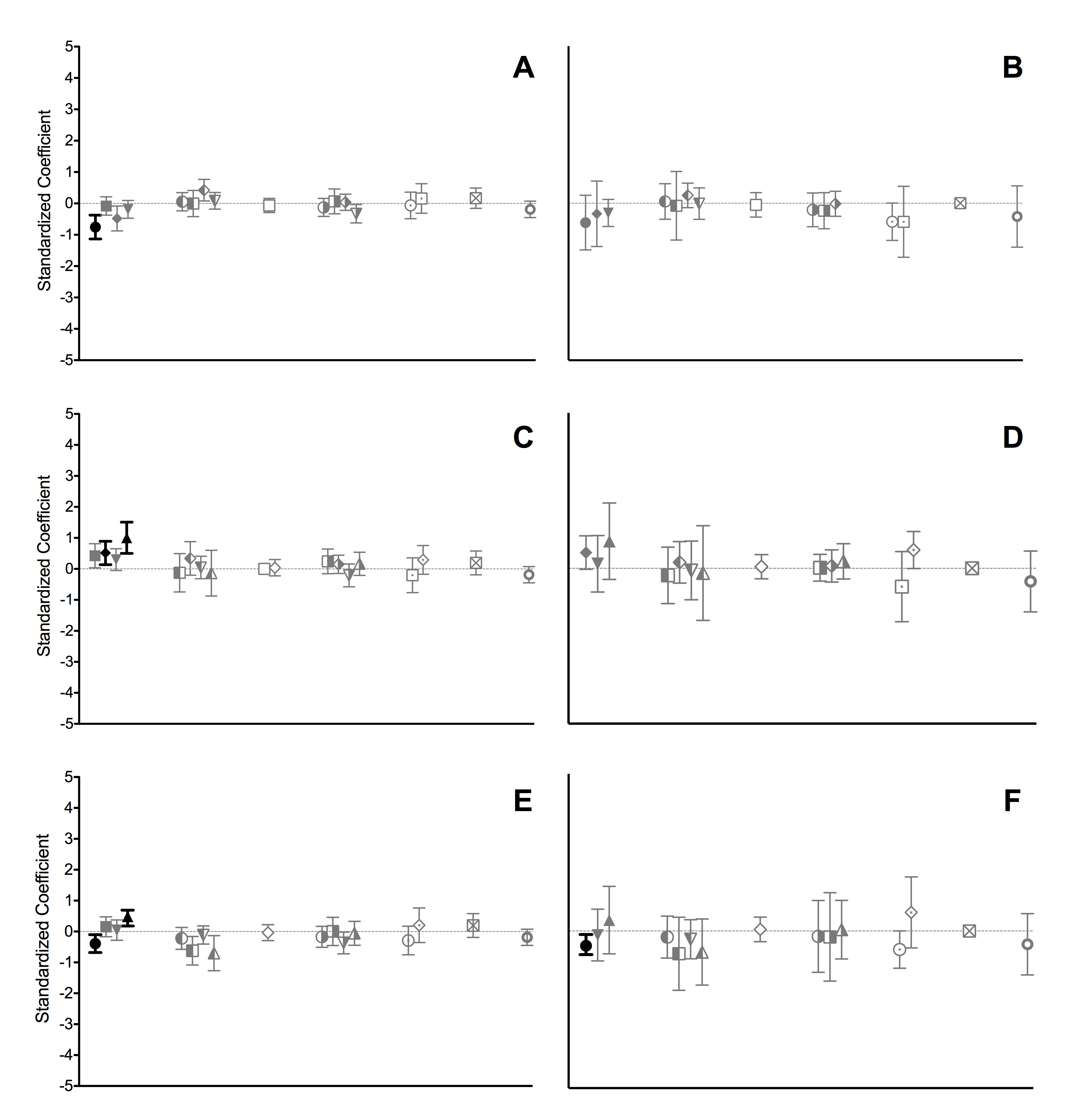

Supplement: Figure S3 — Standardized coefficients for predictors of viral substitution rates based on analyses of control datasets. Standardized coefficients with 95% confidence intervals for the different predictor variables of structural (left) and non-structural (right) gene substitution rates, using the datasets with one rate per viral species. A and B show the coefficients from the first analysis, C and D show coefficients from the second analysis, and E and F show coefficients from the third analysis. Coefficients are indicated by the same symbols used in Figures 1 and 2. Dark coefficients correspond to significant substitution rate predictors (P<0.01, epithelial, leukocyte, hepatocyte, and epithelial target cells in A, leukocyte and epithelial target cells in C, neural and epithelial target cells in E, and neural target cells in F), while the other coefficients are shown in gray. (TIFF) [file ppat.1003838.s003.tiff]
