# Supplementary material for: Cell Tropism Predicts Long-term Nucleotide Substitution Rates of Mammalian RNA Viruses
Source: PLoS Pathog. 2014 Jan 9;10(1):e1003838. doi: 10.1371/journal.ppat.1003838 (PMC3887100; doi:10.1371/journal.ppat.1003838)
Supplement: Table S1 — Nucleotide substitution rates and characteristics of all viruses used in this study. (DOCX) [file ppat.1003838.s004.docx]

**Table S1.** Nucleotide substitution rates and characteristics of all viruses used in this study.

| Virus | Genome Architecture | Family | Genome Length (kb) | Segmented or Non-segmented | Principal Target Cell(s)^a^ | Principal Transmission Route(s) | Infection Duration | Host Range^b^ | Nucleotide Substitution Rate (x10^-3^)^c^ | dN/dS^d^ | Gene^e^ | Source of Rate, dN/dS |
| --- | --- | --- | --- | --- | --- | --- | --- | --- | --- | --- | --- | --- |
| Human astrovirus (hAstV) | (+)ssRNA | *Astroviridae* | 7.3 | Non-segmented | Intestinal epithelial cells [1] | Fecal-oral [1] | Acute, persistent [1] | Species (*Homo sapiens*) [1] | 2.38  (1.43-3.49) | 0.07 | S (p) | This study |
| Equine arteritis virus (EAV) | (+)ssRNA | *Arteriviridae* | 12.7 | Non-segmented | Leukocytes (systemic) [2,3] | Respiratory, vertical [2,3] | Acute, persistent [2,3] | Genus (*Equus*) [3] | 2.70  (2.09-3.28) | 0.27 | S | This study |
| Porcine reproductive and respiratory syndrome virus (PRRSV) | (+)ssRNA | *Arteriviridae* | 15.4 | Non-segmented | Leukocytes (systemic) [2,4,5,6] | Respiratory, vertical [2,4] | Acute, persistent [2,4] | Species  (*Sus scrofa*) [2,4] | 5.19  (1.97-8.23) | 0.14 | NS | This study |
| Porcine reproductive and respiratory syndrome virus type 2 (PRRSV-2) | (+)ssRNA | *Arteriviridae* | 15.4 | Non-segmented | Leukocytes (systemic) [2,4,5,6] | Respiratory, vertical [2,4] | Acute, persistent [2,4] | Species  (*Sus scrofa*) [2,4] | 9.60  (8.70-11.00) | N/A | S | [7] |
| Norwalk virus GII.b (NoV GII.b) | (+)ssRNA | *Caliciviridae* | 7.7 | Non-segmented | Intestinal epithelial cells [8,9] | Fecal-oral [9,10,11,12] | Acute, persistent [9] | Class (Mammalia) [13] | 6.12 | N/A | NS | [14] |
| Norwalk virus GII.3  (NoV GII.3) | (+)ssRNA | *Caliciviridae* | 7.7 | Non-segmented | Intestinal epithelial cells [8,9] | Fecal-oral [9,10,11,12] | Acute, persistent [9] | Class (Mammalia) [13] | 5.80  (4.40-7.40) | N/A | S | [15] |
|  |  |  |  |  |  |  |  |  | 5.54  (4.43-6.74) | N/A | S | [14] |
|  |  |  |  |  |  |  |  |  | 3.99 | N/A | NS | [14] |
| Norwalk virus GII.4 (NoV GII.4) | (+)ssRNA | *Caliciviridae* | 7.7 | Non-segmented | Intestinal epithelial cells [8,9] | Fecal-oral [9,10,11,12] | Acute, persistent [9] | Class (Mammalia) [13] | 5.33  (4.62-6.02) | N/A | S | [16] |
|  |  |  |  |  |  |  |  |  | 5.10  (4.40-6.00) | N/A | S | [17] |
|  |  |  |  |  |  |  |  |  | 5.63  (3.62-7.71) | 0.07 | NS | This study |
| Rabbit hemorrhagic disease virus (RHDV) | (+)ssRNA | *Caliciviridae* | 7.4 | Non-segmented | Leukocytes (systemic) [18,19] | Respiratory [20,21,22] | Acute, persistent [21,22,23] | Family (Leporidae) [20,23,24] | 1.91  (1.50-2.34) | 0.09 | S | [25] |
|  |  |  |  |  |  |  |  |  | 1.92  (1.24-2.49) | 0.07 | NS | [25] |
| Bovine coronavirus (bCoV) | (+)ssRNA | *Coronaviridae* | 31.0 | Non-segmented | Intestinal, respiratory epithelial cells [26,27,28,29] | Fecal-oral, Respiratory [29,30] | Acute, persistent [31] | Family (Bovidae) [27,28,29] | 0.83  (0.52-1.16) | 0.32 | S (p) | This study |
| Dengue virus  (DENV) | (+)ssRNA | *Flaviviridae* | 10.7 | Non-segmented | Leukocytes (systemic) [32,33] | Arthropod vector [32] | Acute [32,33] | Order (Primates) [32,34] | 0.76  (0.66-0.87) | 0.06-0.07 | S | [35] |
| Dengue virus type 1  (DENV-1) | (+)ssRNA | *Flaviviridae* | 10.7 | Non-segmented | Leukocytes (systemic) [32,33] | Arthropod vector [32] | Acute [32,33] | Order (Primates) [32,34] | 0.70  (0.60-0.80) | 0.07 | S | [36] |
|  |  |  |  |  |  |  |  |  | 0.78  (0.65-0.91) | N/A | S | [37] |
|  |  |  |  |  |  |  |  |  | 0.86  (0.65-1.10) | N/A | S | [38] |
| Dengue virus type 2  (DENV-2) | (+)ssRNA | *Flaviviridae* | 10.7 | Non-segmented | Leukocytes (systemic) [32,33] | Arthropod vector [32] | Acute [32,33] | Order (Primates) [32,34] | 0.71  (0.60-0.82) | 0.06 | S | [36] |
|  |  |  |  |  |  |  |  |  | 0.75  (0.63-0.87) | 0.25 | S | [39] |
|  |  |  |  |  |  |  |  |  | 0.65  (0.41-0.87) | N/A | S | [40] |
|  |  |  |  |  |  |  |  |  | 0.83  (0.66-0.98) | 0.05 | S | [41] |
|  |  |  |  |  |  |  |  |  | 0.80  (0.66-0.95) | N/A | S | [42] |
|  |  |  |  |  |  |  |  |  | 0.86  (0.74-0.98) | N/A | S | [37] |
|  |  |  |  |  |  |  |  |  | 0.85  (0.70-1.00) | N/A | S | [38] |
|  |  |  |  |  |  |  |  |  | 0.69  (0.59-0.79) | 0.05 | NS | [41] |
| Dengue virus type 3  (DENV-3) | (+)ssRNA | *Flaviviridae* | 10.7 | Non-segmented | Leukocytes (systemic) [32,33] | Arthropod vector [32] | Acute [32,33] | Order (Primates) [32,34] | 0.87  (0.76-0.98) | 0.08 | S | [36] |
|  |  |  |  |  |  |  |  |  | 0.90  (0.69-1.00) | N/A | S | [43] |
|  |  |  |  |  |  |  |  |  | 1.10  (0.83-1.38) | N/A | S | [44] |
|  |  |  |  |  |  |  |  |  | 0.95  (0.73-1.04) | N/A | S | [37] |
|  |  |  |  |  |  |  |  |  | 0.87  (0.67-1.08) | N/A | S | [38] |
|  |  |  |  |  |  |  |  |  | 0.89  (0.79-1.00) | N/A | S | [45] |
| Dengue virus type 4  (DENV-4) | (+)ssRNA | *Flaviviridae* | 10.7 | Non-segmented | Leukocytes (systemic) [32,33] | Arthropod vector [32] | Acute [32,33] | Order (Primates) [32,34] | 0.69  (0.41-1.00) | N/A | S | [43] |
|  |  |  |  |  |  |  |  |  | 0.97  (0.79-1.06) | N/A | S | [37] |
|  |  |  |  |  |  |  |  |  | 0.06  (0.05-0.08) | N/A | S | [38] |
|  |  |  |  |  |  |  |  |  | 0.72  (0.58-0.88) | 0.07 | S | [36] |
|  |  |  |  |  |  |  |  |  | 0.83  (0.68-1.00) | N/A | S | [42] |
| Japanese encephalitis virus (JEV) | (+)ssRNA | *Flaviviridae* | 11.0 | Non-segmented | Neurons [32,46,47] | Arthropod vector [32] | Acute, persistent [32,46,47] | Class (Mammalia) [32] | 0.14  (0.09-0.20) | 0.16 | S | [48] |
|  |  |  |  |  |  |  |  |  | 1.18  (0.72-1.40) | 0.02 | S | [48] |
|  |  |  |  |  |  |  |  |  | 0.15  (0.07-0.24) | 0.05 | NS | This study |
| Tick-borne encephalitis virus (TBEV) | (+)ssRNA | *Flaviviridae* | 11.1 | Non-segmented | Neurons [32,49] | Arthropod vector [32] | Acute, persistent [32] | Order (Rodentia) [32,34] | 0.14 | 0.05 | S | [50] |
|  |  |  |  |  |  |  |  |  | 0.79  (0.41-1.12) | N/A | S | [51] |
|  |  |  |  |  |  |  |  |  | 0.03  (0.01-0.07) | N/A | S (p) | [52] |
|  |  |  |  |  |  |  |  |  | 0.02  (0.00-0.04) | 0.06 | NS | This study |
| Powassan virus (POWV) | (+)ssRNA | *Flaviviridae* | 10.8 | Non-segmented | Neurons [32,53] | Arthropod vector [32] | Acute [32,53] | Class (Mammalia) [32,34,53] | 0.34  (0.09-0.68) | 0.09 | S | This study |
| Yellow fever virus (YFV) | (+)ssRNA | *Flaviviridae* | 10.9 | Non-segmented | Leukocytes (systemic) [32,54] | Arthropod vector [32] | Acute [32,54] | Order (Primates) [32,34] | 0.21  (0.10-0.33) | 0.04 | S | [36] |
|  |  |  |  |  |  |  |  |  | 0.13  (0.05-0.21) | 0.05 | NS | This study |
| Hepatitis C virus type 1a (HCV1a) | (+)ssRNA | *Flaviviridae* | 9.5 | Non-segmented | Hepatocytes [55] | Parenteral [55] | Acute, persistent [55] | Species (*Homo sapiens*) [55] | 2.45  (1.67-3.21) | 0.27 | S | [56]^f^ |
|  |  |  |  |  |  |  |  |  | 3.41  (2.54-4.32) | N/A | S (p) | [57] |
|  |  |  |  |  |  |  |  |  | 0.71  (0.38-1.07) | 0.11 | NS | [56]^f^ |
| Hepatitis C virus type 1b (HCV1b) | (+)ssRNA | *Flaviviridae* | 9.5 | Non-segmented | Hepatocytes [55] | Parenteral [55] | Acute, persistent [55] | Species (*Homo sapiens*) [55] | 2.72  (1.71-3.75) | 0.26 | S | [56]^f^ |
|  |  |  |  |  |  |  |  |  | 0.57  (0.22-0.96) | 0.12 | NS | [56]^f^ |
| Hepatitis E virus (HEV) | (+)ssRNA | *Hepeviridae* | 7.2 | Non-segmented | Hepatocytes [58] | Fecal-oral [58,59] | Acute [58] | Class (Mammalia) [58] | 1.13 | N/A | S | [60] |
| Foot-and-mouth disease virus (FMDV) | (+)ssRNA | *Picornaviridae* | 8.2 | Non-segmented | Many epithelial cells [61] | Respiratory [62] | Acute, persistent [62,63] | Order (Artiodactyla) [62] | 2.48  (1.69-3.31) | N/A | S | [64] |
| Foot-and-mouth disease virus type A  (FMDV-A) | (+)ssRNA | *Picornaviridae* | 8.2 | Non-segmented | Many epithelial cells [61] | Respiratory [62] | Acute, persistent [62,63] | Order (Artiodactyla) [62] | 5.77  (4.81-6.74) | N/A | S | [65] |
|  |  |  |  |  |  |  |  |  | 1.45  (0.07-2.24) | 0.05 | NS | [66] |
| Foot-and-mouth disease virus type O  (FMDV-O) | (+)ssRNA | *Picornaviridae* | 8.2 | Non-segmented | Many epithelial cells [61] | Respiratory [62] | Acute, persistent [62,63] | Order (Artiodactyla) [62] | 4.81  (4.04-5.46) | N/A | S | [65] |
| Foot-and-mouth disease virus type SAT2  (FMDV-SAT2) | (+)ssRNA | *Picornaviridae* | 8.2 | Non-segmented | Many epithelial cells [61] | Respiratory [62] | Acute, persistent [62,63] | Order (Artiodactyla) [62] | 2.42  (1.75-3.12) | N/A | S | [67] |
| Coxsackievirus A16 (CVA16) | (+)ssRNA | *Picornaviridae* | 7.4 | Non-segmented | Many epithelial cells [68,69,70,71,72] | Fecal-oral  [68,70,71,73] | Acute [68] | Species (*Homo sapiens*) [68,71] | 5.38  (3.96-6.85) | 0.04 | S | This study |
|  |  |  |  |  |  |  |  |  | 6.23  (4.46-8.11) | 0.05 | NS (p) | This study |
| Enterovirus 71 (EV71) | (+)ssRNA | *Picornaviridae* | 7.4 | Non-segmented | Many epithelial cells [68,69,70,71,72] | Fecal-oral [68,70,71,73] | Acute [68] | Species (*Homo sapiens*) [68,71] | 5.53  (4.29-6.67) | 0.05 | NS | [66] |
| Enterovirus 71 type B (EV71-B) | (+)ssRNA | *Picornaviridae* | 7.4 | Non-segmented | Many epithelial cells [68,69,70,71,72] | Fecal-oral [68,70,71,73] | Acute [68] | Species (*Homo sapiens*) [68,71] | 4.50  (4.30-4.70) | N/A | S | [74] |
| Enterovirus 71 type C (EV71-C) | (+)ssRNA | *Picornaviridae* | 7.4 | Non-segmented | Many epithelial cells [68,69,70,71,72] | Fecal-oral [68,70,71,73] | Acute [68] | Species (*Homo sapiens*) [68,71] | 3.66  (3.25-4.05) | N/A | S | [75] |
|  |  |  |  |  |  |  |  |  | 4.20  (4.00-4.40) | N/A | S | [74] |
| Coxsackievirus B3 (CVB3) | (+)ssRNA | *Picornaviridae* | 7.4 | Non-segmented | Intestinal epithelial cells [68,69,70,71,72] | Fecal-oral [68,70,71,73] | Acute, persistent [68,76,77,78,79] | Species (*Homo sapiens*) [68,71] | 4.80  (3.80-5.80) | N/A | S | [80] |
| Coxsackievirus B4 (CVB4) | (+)ssRNA | *Picornaviridae* | 7.4 | Non-segmented | Intestinal epithelial cells [68,69,70,71,72] | Fecal-oral [68,70,71,73] | Acute, persistent [68] | Species (*Homo sapiens*) [68,71] | 4.95  (4.17-5.83) | 0.02 | S | This study |
| Coxsackievirus B5 (CVB5) | (+)ssRNA | *Picornaviridae* | 7.4 | Non-segmented | Intestinal epithelial cells [68,69,70,71,72] | Fecal-oral [68,70,71,73] | Acute, persistent [68] | Species (*Homo sapiens*) [68,71] | 4.20  (3.30-5.20) | N/A | S | [81] |
| Echovirus 6 (E6) | (+)ssRNA | *Picornaviridae* | 7.4 | Non-segmented | Intestinal, respiratory epithelial cells [68,69,70,71,72] | Fecal-oral [68,70,71,73] | Acute [68] | Species (*Homo sapiens*) [68,71] | 6.42  (5.04-7.84) | 0.04 | S | This study |
| Echovirus 9 (E9) | (+)ssRNA | *Picornaviridae* | 7.4 | Non-segmented | Intestinal, respiratory epithelial cells [68,69,70,71,72] | Fecal-oral [68,70,71,73] | Acute [68] | Species (*Homo sapiens*) [68,71] | 5.80  (3.70-8.10) | N/A | S | [82] |
|  |  |  |  |  |  |  |  |  | 9.37  (5.17-14.34) | 0.03 | NS (p) | This study |
| Echovirus 11 (E11) | (+)ssRNA | *Picornaviridae* | 7.4 | Non-segmented | Intestinal, respiratory epithelial cells [68,69,70,71,72] | Fecal-oral [68,70,71,73] | Acute [68] | Species (*Homo sapiens*) [68,71] | 4.80  (3.60-6.10) | N/A | S | [82] |
|  |  |  |  |  |  |  |  |  | 4.30  (1.66-7.42) | 0.03 | NS (p) | This study |
| Echovirus 13 (E13) | (+)ssRNA | *Picornaviridae* | 7.4 | Non-segmented | Intestinal, respiratory epithelial cells [68,69,70,71,72] | Fecal-oral [68,70,71,73] | Acute [68] | Species (*Homo sapiens*) [68,71] | 15.01  (7.52-24.98) | 0.04 | S | This study |
| Echovirus 30 (E30) | (+)ssRNA | *Picornaviridae* | 7.4 | Non-segmented | Intestinal, respiratory epithelial cells [68,69,70,71,72] | Fecal-oral [68,70,71,73] | Acute [68] | Species (*Homo sapiens*) [68,71] | 4.38  (3.95-4.83) | 0.06 | S | This study |
|  |  |  |  |  |  |  |  |  | 4.30  (1.66-7.42) | 0.03 | NS (p) | This study |
| Echovirus 33 (E33) | (+)ssRNA | *Picornaviridae* | 7.4 | Non-segmented | Intestinal, respiratory epithelial cells [68,69,70,71,72] | Fecal-oral [68,70,71,73] | Acute [68] | Species (*Homo sapiens*) [68,71] | 10.71  (5.48-15.57) | 0.04 | S | This study |
| Swine vesicular disease virus (SVDV) | (+)ssRNA | *Picornaviridae* | 7.4 | Non-segmented | Many epithelial cells [83] | Fecal-oral [84] | Acute, persistent [84,85] | Genus  (*Sus*) [83] | 3.49  (2.44-4.56) | 0.10 | S | This study |
| Coxsackievirus A24 (CVA24) | (+)ssRNA | *Picornaviridae* | 7.4 | Non-segmented | Intestinal epithelial cells [68,69,70,71,72] | Fecal-oral [68,70,71,73] | Acute, persistent [68] | Species (*Homo sapiens*) [68,71] | 11.82  (9.23-14.57) | 0.07 | S | This study |
|  |  |  |  |  |  |  |  |  | 11.61  (8.73-14.44) | 0.06 | NS  (3C) | This study |
| Poliovirus type 1 (PV1) | (+)ssRNA | *Picornaviridae* | 7.4 | Non-segmented | Intestinal epithelial cells [68,69,70,71,72] | Fecal-oral [68,70,71,73] | Acute, persistent [68] | Species (*Homo sapiens*) [68,71] | 6.56  (5.96-7.15) | 0.04 | S | This study |
|  |  |  |  |  |  |  |  |  | 11.60  (3.52-19.87) | 0.02 | NS (p) | This study |
| Enterovirus 68 (EV68) | (+)ssRNA | *Picornaviridae* | 7.4 | Non-segmented | Intestinal epithelial cells [68,69,70,71,72] | Fecal-oral [68,70,71,73] | Acute [68] | Species (*Homo sapiens*) [68,71] | 6.20  (5.40-7.10) | N/A | S | [86] |
|  |  |  |  |  |  |  |  |  | 4.93  (4.01-5.85) | 0.09 | S (p) | [87] |
| Hepatitis A virus (HAV) | (+)ssRNA | *Picornaviridae* | 7.5 | Non-segmented | Hepatocytes [88] | Fecal-oral [88] | Acute [88] | Order (Primates) [88] | 1.08  (0.80-1.34) | 0.05 | S | This study |
|  |  |  |  |  |  |  |  |  | 0.36  (0.21-0.52) | 0.04 | NS | This study |
| Human parechovirus (HPeV) | (+)ssRNA | *Picornaviridae* | 7.3 | Non-segmented | Intestinal, respiratory epithelial cells [89] | Fecal-oral, respiratory [89,90] | Acute [89,90] | Species (*Homo sapiens*) [89,90] | 2.79  (2.05-3.66) | N/A | S | [91] |
|  |  |  |  |  |  |  |  |  | 2.96  (1.88-3.92) | 0.04 | NS (p) | [66] |
| Porcine teschovirus (PTV) | (+)ssRNA | *Picornaviridae* | 7.1 | Non-segmented | Leukocytes (systemic) [83,92,93] | Fecal-oral [94] | Acute, persistent [95] | Genus  (*Sus*) [94] | 2.46  (2.03-2.95) | N/A | S | [94] |
|  |  |  |  |  |  |  |  |  | 1.60  (1.34-1.85) | N/A | S | [96] |
|  |  |  |  |  |  |  |  |  | 1.62  (0.63-2.75) | 0.10 | S (p) | [66] |
| Chikungunya virus (CHIKV) | (+)ssRNA | *Togaviridae* | 11.5 | Non-segmented | Leukocytes (systemic) [97,98] | Arthropod vector [99] | Acute, persistent [97,98] | Order (Primates) [99] | 0.84  (0.50-1.23) | N/A | S (p) | [100] |
|  |  |  |  |  |  |  |  |  | 0.66  (0.47-0.87) | 0.07 | NS | This study |
| Ross River virus (RRV) | (+)ssRNA | *Togaviridae* | 11.7 | Non-segmented | Leukocytes (systemic) [99,101] | Arthropod vector [99] | Acute, persistent [99,101] | Class (Mammalia) [99,101] | 0.49  (0.24-0.75) | 0.38 | S | This study |
| Venezuelan equine encephalitis virus (VEEV) | (+)ssRNA | *Togaviridae* | 11.4 | Non-segmented | Neurons [99,102,103] | Arthropod vector [99] | Acute [99,104] | Class (Mammalia) [99,105,106,107] | 0.07  (0.00-0.21) | 0.07 | S | This study |
|  |  |  |  |  |  |  |  |  | 0.12  (0.04-0.21) | 0.04 | NS | This study |
| Western equine encephalitis virus (WEEV) | (+)ssRNA | *Togaviridae* | 11.5 | Non-segmented | Neurons [99,108] | Arthropod vector [99] | Acute, persistent [99,109] | Class (Mammalia) [99,105,110] | 0.17  (0.10-0.26) | 0.23 | S | This study |
| Rubella virus (RuV) | (+)ssRNA | *Togaviridae* | 9.8 | Non-segmented | Leukocytes (systemic) [111,112,113,114] | Respiratory, vertical [115] | Acute, persistent [115] | Species (*Homo sapiens*) [115] | 0.82  (0.68-0.97) | 0.05 | S | This study |
| Lassa virus (LasV) | (-)ssRNA | *Arenaviridae* | 10.7 | Segmented | Leukocytes (systemic) [114,116,117] | Respiratory, vertical [116] | Acute, persistent [116] | Genus (*Mastomys*) [116] | 2.88  (1.41-4.52) | 0.07 | S (p) | This study |
| Lymphocytic choriomeningitis virus (LCMV) | (-)ssRNA | *Arenaviridae* | 10.1 | Segmented | Leukocytes (systemic) [114,116] | Respiratory, vertical [116] | Acute, persistent [116] | Genus  (*Mus*) [116] | 0.33  (0.14-0.52) | N/A | S | [118] |
| Borna disease virus (BDV) | (-)ssRNA | *Bornaviridae* | 8.9 | Non-segmented | Neurons [119,120] | Respiratory [119,121,122,123] | Acute, persistent [119] | Class (Mammalia) [119,121] | 0.08  (0.01-0.15) | 0.03 | S  (N) | This study |
| Dobrava-Belgrade virus (DBV) | (-)ssRNA | *Bunyaviridae* | 11.8 | Segmented | Endothelial cells [124,125] | Fecal-oral, respiratory [125] | Persistent [125] | Genus (*Apodemus*) [125,126] | 0.28  (0.01-0.68) | N/A | S (p)  (N) | [127] |
| Puumala virus (PUUV) | (-)ssRNA | *Bunyaviridae* | 12.1 | Segmented | Endothelial cells [124,125] | Fecal-oral, respiratory [125] | Persistent [125] | Family (Cricetidae) [125,126] | 0.54  (0.07-0.98) | N/A | S  (N) | [127] |
| Seoul virus (SEOV) | (-)ssRNA | *Bunyaviridae* | 12.0 | Segmented | Endothelial cells [124,125] | Fecal-oral, respiratory [125] | Persistent [125] | Genus (*Rattus*) [125,126] | 0.41  (0.02-1.05) | 0.06 | S  (N) | This study |
| Crimean-Congo hemorrhagic fever virus (CCHFV) | (-)ssRNA | *Bunyaviridae* | 19.1 | Segmented | Leukocytes (systemic) [128] | Arthropod vector [125] | Acute [125] | Class (Mammalia) [125] | 0.15  (0.06-0.24) | N/A | S | [129] |
|  |  |  |  |  |  |  |  |  | 0.07  (0.03-0.11) | 0.05 | NS | This study |
| Rift Valley fever virus (RVFV) | (-)ssRNA | *Bunyaviridae* | 12.0 | Segmented | Leukocytes (systemic) [125,130,131] | Arthropod vector [125,131] | Acute, persistent [125] | Class (Mammalia) [125,131] | 0.24  (0.18-0.30) | 0.04-0.07 | S | [132] |
|  |  |  |  |  |  |  |  |  | 0.36  (0.26-0.46) | N/A | S | [133] |
|  |  |  |  |  |  |  |  |  | 0.28  (0.20-0.35) | 0.03 | NS | [132] |
|  |  |  |  |  |  |  |  |  | 0.28  (0.18-0.39) | N/A | NS | [133] |
| Toscana virus (TosV) | (-)ssRNA | *Bunyaviridae* | 12.5 | Segmented | Neurons | Arthropod vector | Acute | Class (Mammalia) | 0.09  (0.00-0.25) | N/A | S | [134] |
|  |  |  |  |  |  |  |  |  | 0.25  (0.03-0.54) | N/A | S (p) | [135] |
| Influenza A virus (FLUAV) | (-)ssRNA | *Orthomyxoviridae* | 13.6 | Segmented | Respiratory epithelial cells [136,137] | Respiratory [136,137] | Acute [136,137] | Class (Mammalia) [136,137] | 3.92  (2.43-5.40) | 0.13 | S | [138] |
|  |  |  |  |  |  |  |  |  | 2.23  (1.98-2.49) | N/A | S | [139] |
|  |  |  |  |  |  |  |  |  | 3.00  (2.50-3.40) | 0.03 | NS^g^ | [140] |
|  |  |  |  |  |  |  |  |  | 2.86  (1.93-3.75) | 0.05 | NS^g^ | [138] |
|  |  |  |  |  |  |  |  |  | 2.10  (1.90-2.31) | N/A | NS^g^ | [139] |
|  |  |  |  |  |  |  |  |  | 2.60  (2.29-2.92) | N/A | NS^g^ | [141] |
|  |  |  |  |  |  |  |  |  | 2.59  (2.13-3.04) | N/A | NS^g^ | [142] |
| Influenza A virus H1 (FLUAV H1) | (-)ssRNA | *Orthomyxoviridae* | 13.6 | Segmented | Respiratory epithelial cells [136,137] | Respiratory [136,137] | Acute [136,137] | Class (Mammalia) [136,137] | 3.67  (3.41-3.92) | N/A | S | [141] |
| Influenza A virus H4 (FLUAV H4) | (-)ssRNA | *Orthomyxoviridae* | 13.6 | Segmented | Respiratory epithelial cells [136,137] | Respiratory [136,137] | Acute [136,137] | Class (Mammalia) [136,137] | 2.50  (2.00-3.10) | 0.09 | S | [140] |
| Influenza A virus H5 (FLUAV H5) | (-)ssRNA | *Orthomyxoviridae* | 13.6 | Segmented | Respiratory epithelial cells [136,137] | Respiratory [136,137] | Acute [136,137] | Class (Mammalia) [136,137] | 4.77  (3.88-5.74) | 0.27 | S | [142] |
| Influenza A virus H6 (FLUAV H6) | (-)ssRNA | *Orthomyxoviridae* | 13.6 | Segmented | Respiratory epithelial cells [136,137] | Respiratory [136,137] | Acute [136,137] | Class (Mammalia) [136,137] | 4.20  (3.30-5.00) | 0.15 | S | [140] |
| Influenza B virus (FLUBV) | (-)ssRNA | *Orthomyxoviridae* | 13.6 | Segmented | Respiratory epithelial cells [136,137] | Respiratory [136,137] | Acute [136,137] | Class (Mammalia) [136,137] | 2.15  (1.85-2.46) | 0.22 | S | [143] |
|  |  |  |  |  |  |  |  |  | 0.27  (0.12-0.41) | 0.04 | NS^g^ | [143] |
| Influenza C virus (FLUCV) | (-)ssRNA | *Orthomyxoviridae* | 12.6 | Segmented | Respiratory epithelial cells [136,137] | Respiratory [136,137] | Acute [136,137] | Class (Mammalia) [136,137] | 0.49  (0.41-0.57) | N/A | S | [144] |
|  |  |  |  |  |  |  |  |  | 0.68  (0.48-0.89) | N/A | NS^g^ | [144] |
| Canine distemper virus (CDV) | (-)ssRNA | *Paramyxoviridae* | 15.7 | Non-segmented | Leukocytes (systemic) [114,145,146] | Respiratory [145,146] | Acute, persistent [145,147] | Class (Mammalia) [145,146] | 1.05  (0.52-1.60) | 0.26 | S | [148] |
| Measles virus (MeV) | (-)ssRNA | *Paramyxoviridae* | 15.9 | Non-segmented | Leukocytes (systemic) [114,146,149] | Respiratory [146,149] | Acute, persistent [146,149] | Species (*Homo sapiens*) [146,149] | 0.66  (0.48-0.83) | 0.23 | S | [150] |
|  |  |  |  |  |  |  |  |  | 0.56  (0.45-0.68) | 0.02 | S (p) | [151] |
|  |  |  |  |  |  |  |  |  | 0.42  (0.27-0.56) | 0.52 | NS  (P/C/V) | This study |
| Human parainfluenza virus 1 (HPiV-1) | (-)ssRNA | *Paramyxoviridae* | 15.6 | Non-segmented | Respiratory epithelial cells [152] | Respiratory [152] | Acute [152] | Species (*Homo sapiens*) [152] | 1.37  (1.16-1.59) | N/A | S | [153] |
| Mumps virus (MuV) | (-)ssRNA | *Paramyxoviridae* | 15.4 | Non-segmented | Leukocytes (systemic) [154,155] | Respiratory [155] | Acute, persistent [155] | Species (*Homo sapiens*) [155] | 0.41  (0.30-0.51) | 0.16 | S | This study |
| Human metapneumovirus (HMPV) | (-)ssRNA | *Paramyxoviridae* | 13.3 | Non-segmented | Respiratory epithelial cells [156,157] | Respiratory [157] | Acute, persistent [156] | Species (*Homo sapiens*) [157] | 7.40  (5.72-9.15) | N/A | S | [158] |
|  |  |  |  |  |  |  |  |  | 3.50  (2.30-4.80) | 0.52 | S | [159] |
|  |  |  |  |  |  |  |  |  | 5.18  (3.76-6.78) | N/A | S | [160] |
|  |  |  |  |  |  |  |  |  | 6.49  (4.60-8.44) | N/A | S | [160] |
| Human respiratory syncytial virus type A (HRSV-A) | (-)ssRNA | *Paramyxoviridae* | 15.2 | Non-segmented | Respiratory epithelial cells [157,161] | Respiratory [157] | Acute, persistent [157] | Species (*Homo sapiens*) [157] | 2.22  (1.93-2.56) | N/A | S | [162] |
| Bovine ephemeral fever virus (BEFV) | (-)ssRNA | *Rhabdoviridae* | 14.9 | Non-segmented | Leukocytes (systemic) [163,164,165,166] | Arthropod vector [166] | Acute  [163,165,166] | Family (Bovinae) [165,166] | 0.87  (0.48-1.28) | 0.10 | S | This study |
| Rabies virus (RabV) | (-)ssRNA | *Rhabdoviridae* | 11.9 | Non-segmented | Neurons [166] | Bites, scratches [166] | Acute [166] | Class (Mammalia) [166] | 0.39  (0.12-0.65) | N/A | S | [167] |
|  |  |  |  |  |  |  |  |  | 0.40  (0.21-0.60) | N/A | S | [168] |
|  |  |  |  |  |  |  |  |  | 0.33  (0.22-0.43) | 0.08 | S | [169] |
|  |  |  |  |  |  |  |  |  | 0.32  (0.22-0.44) | N/A | S | [170] |
|  |  |  |  |  |  |  |  |  | 0.63  (0.33-1.10) | N/A | S | [171] |
|  |  |  |  |  |  |  |  |  | 0.09  (0.00-0.20) | 0.04 | NS (p) | This study |
| European Bat Lyssavirus 1  (EBLV-1) | (-)ssRNA | *Rhabdoviridae* | 12.0 | Non-segmented | Neurons [172,173] | Bites, scratches [166,174] | Acute [172,173] | Order (Chiroptera) [172,173] | 0.05  (0.00-0.09) | N/A | S | [175] |
| Bluetongue virus (BTV) | dsRNA | *Reoviridae* | 19.2 | Segmented | Leukocytes (systemic) [176,177] | Arthropod vector [176,177] | Acute, persistent [176,177] | Class (Mammalia) [176,177] | 0.49  (0.20-0.81) | 0.12 | S | [178] |
|  |  |  |  |  |  |  |  |  | 0.69  (0.34-1.07) | 0.06 | NS  (NS3) | [178] |
| Epizootic hemorrhagic disease virus type 2  (EHDV-2) | dsRNA | *Reoviridae* | 19.4 | Segmented | Leukocytes (systemic) [177,179,180] | Arthropod vector [177,181] | Acute, persistent [177,179] | Class (Mammalia) [177,181] | 0.48  (0.31-0.66) | N/A | NS (p)  (NS3) | [181] |
| Rotavirus A (RVA) | dsRNA | *Reoviridae* | 18.6 | Segmented | Intestinal epithelial cells [182] | Fecal-oral [182] | Acute, persistent [182] | Class (Mammalia) [182] | 2.49  (1.00-4.08) | 0.03 | NS | This study |
| Rotavirus A G1  (RVA G1) | dsRNA | *Reoviridae* | 18.6 | Segmented | Intestinal epithelial cells [182] | Fecal-oral [182] | Acute, persistent [182] | Class (Mammalia) [182] | 1.41  (1.03-1.79) | N/A | S | [183] |
| Rotavirus A G2  (RVA G2) | dsRNA | *Reoviridae* | 18.6 | Segmented | Intestinal epithelial cells [182] | Fecal-oral [182] | Acute, persistent [182] | Class (Mammalia) [182] | 1.53  (0.80-2.38) | 0.17 | S | This study |
| Rotavirus A G3  (RVA G3) | dsRNA | *Reoviridae* | 18.6 | Segmented | Intestinal epithelial cells [182] | Fecal-oral [182] | Acute, persistent [182] | Class (Mammalia) [182] | 1.95  (1.55-2.39) | 0.19 | S | This study |
| Rotavirus A G9  (RVA G9) | dsRNA | *Reoviridae* | 18.6 | Segmented | Intestinal epithelial cells [182] | Fecal-oral [182] | Acute, persistent [182] | Class (Mammalia) [182] | 1.87  (1.45-2.27) | N/A | S | [184] |
| Rotavirus A G12  (RVA G12) | dsRNA | *Reoviridae* | 18.6 | Segmented | Intestinal epithelial cells [182] | Fecal-oral [182] | Acute, persistent [182] | Class (Mammalia) [182] | 1.66  (1.13-2.32) | N/A | S | [184] |
| Rotavirus B (RVB) | dsRNA | *Reoviridae* | 18.0 | Segmented | Intestinal epithelial cells [182] | Fecal-oral [182] | Acute, persistent [182] | Class (Mammalia) [182] | 1.38  (0.36-2.72) | 0.10 | S | [185] |
|  |  |  |  |  |  |  |  |  | 1.91  (0.59-3.41) | 0.32-0.23 | NS  (NSP1) | [185] |
| Rotavirus C (RVC) | dsRNA | *Reoviridae* | 17.9 | Segmented | Intestinal epithelial cells [182] | Fecal-oral [182] | Acute, persistent [182] | Class (Mammalia) [182] | 10.24  (7.36-13.05) | 0.09 | S | This study |
|  |  |  |  |  |  |  |  |  | 13.04  (7.15-36.03) | 0.13 | NS (p)  (NSP4) | This study |

^a^Principal target cell(s) refers to the cell(s) that is/are most commonly targeted by the virus. Viruses that infect leukocytes as well as a variety of different cell types (with no clear principal target cell) are classified as “leukocytes (systemic)”.

^b^Some host ranges classified as class-wide (infecting mammals from different orders) may be broader (*i.e.*, also infect non-mammalian vertebrate hosts, or, in the case of arboviruses, arthropod hosts).

^c^Substitution rates are given in nucleotide substitutions/site/year; 95% HPD shown in parentheses, when available.

^d^N/A indicates that dN/dS ratios were not calculated, were calculated by methods other than the Single Likelihood Ancestor Counting method, or were only reported for individual codon positions and/or individual lineages within the full dataset.

^e^Unless other otherwise indicated, S refers to the outer structural protein with the major antigenic site(s), and NS refers to the RNA-dependent RNA polymerase. (p) indicates partial gene sequences.

^f^Rates in this paper were reported for small partitions of the full genome that did not coincide with gene boundaries. The single-gene rates (and dN/dS ratios) reported here were produced in this study using the exact datasets from the paper cited.

^g^The PB1 polymerase gene was used for all of the influenza viruses.

References

1. Mendez E, Arias CF (2007) Astroviruses. In: Knipe DM, Howley PM, Griffin DE, Lamb RA, Martin MA et al., editors. Fields Virology. 5th ed. Philadelphia, PA: Lippincott Williams & Wilkins. pp. 981-1000.

2. Snijder EJ, Spaan WJ (2007) Arteriviruses. In: Knipe DM, Howley PM, Griffin DE, Lamb RA, Martin MA et al., editors. Fields Virology. 5th ed. Philadelphia, PA: Lippincott Williams & Wilkins. pp. 1337-1355.

3. Balasuriya UB, Hedges JF, Smalley VL, Navarrette A, McCollum WH, et al. (2004) Genetic characterization of equine arteritis virus during persistent infection of stallions. Journal of General Virology 85: 379-390.

4. Zimmerman JJ, Yoon KJ, Pirtle EC, Wills RW, Sanderson TJ, et al. (1997) Studies of porcine reproductive and respiratory syndrome (PRRS) virus infection in avian species. Veterinary Microbiology 55: 329-336.

5. Duan X, Nauwynck HJ, Pensaert MB (1997) Virus quantification and identification of cellular targets in the lungs and lymphoid tissues of pigs at different time intervals after inoculation with porcine reproductive and respiratory syndrome virus (PRRSV). Veterinary Microbiology 56: 9-19.

6. Rowland RR, Lawson S, Rossow K, Benfield DA (2003) Lymphoid tissue tropism of porcine reproductive and respiratory syndrome virus replication during persistent infection of pigs originally exposed to virus in utero. Veterinary Microbiology 96: 219-235.

7. Shi M, Lam TTY, Hon CC, Murtaugh MP, Davies PR, et al. (2010) Phylogeny-Based Evolutionary, Demographical, and Geographical Dissection of North American Type 2 Porcine Reproductive and Respiratory Syndrome Viruses. Journal of Virology 84: 8700-8711.

8. Karst SM (2010) Pathogenesis of noroviruses, emerging RNA viruses. Viruses 2: 748-781.

9. Green KY (2007) *Caliciviridae*: The noroviruses. In: Knipe DM, Howley PM, Griffin DE, Lamb RA, Martin MA et al., editors. Fields Virology. 5th ed. Philadelphia, PA: Lippincott Williams & Wilkins. pp. 949-979.

10. Donaldson EF, Lindesmith LC, Lobue AD, Baric RS (2008) Norovirus pathogenesis: mechanisms of persistence and immune evasion in human populations. Immunol Rev 225: 190-211.

11. Moe CL (2009) Preventing norovirus transmission: how should we handle food handlers? Clin Infect Dis 48: 38-40.

12. Parashar U, Quiroz ES, Mounts AW, Monroe SS, Fankhauser RL, et al. (2001) "Norwalk-like viruses". Public health consequences and outbreak management. MMWR Recomm Rep 50: 1-17.

13. Widdowson MA, Monroe SS, Glass RI (2005) Are noroviruses emerging? Emerg Infect Dis 11: 735-737.

14. Mahar JE, Bok K, Green KY, Kirkwood CD (2013) The Importance of Intergenic Recombination in Norovirus GII.3 Evolution. Journal of Virology 87: 3687-3698.

15. Boon D, Mahar JE, Abente EJ, Kirkwood CD, Purcell RH, et al. (2011) Comparative Evolution of GII.3 and GII.4 Norovirus over a 31-Year Period. Journal of Virology 85: 8656-8666.

16. Siebenga JJ, Lemey P, Pond SLK, Rambaut A, Vennema H, et al. (2010) Phylodynamic Reconstruction Reveals Norovirus GII. 4 Epidemic Expansions and their Molecular Determinants. Plos Pathogens 6.

17. Bok K, Prikhodko VG, Green KY, Sosnovtsev SV (2009) Apoptosis in Murine Norovirus-Infected RAW264.7 Cells Is Associated with Downregulation of Survivin. Journal of Virology 83: 3647-3656.

18. Ramiro-Ibanez F, Martin-Alonso JM, Garcia Palencia P, Parra F, Alonso C (1999) Macrophage tropism of rabbit hemorrhagic disease virus is associated with vascular pathology. Virus Res 60: 21-28.

19. Kimura T, Mitsui I, Okada Y, Furuya T, Ochiai K, et al. (2001) Distribution of rabbit haemorrhagic disease virus RNA in experimentally infected rabbits. J Comp Pathol 124: 134-141.

20. Le Gall G, Arnauld C, Boilletot E, Morisse JP, Rasschaert D (1998) Molecular epidemiology of rabbit haemorrhagic disease virus outbreaks in France during 1988 to 1995. Journal of General Virology 79 ( Pt 1): 11-16.

21. Cooke BD, Fenner F (2002) Rabbit haemorrhagic disease and the biological control of wild rabbits, Oryctolagus cuniculus, in Australia and New Zealand. Wildlife Research 29: 689-706.

22. Cooke BD (2002) Rabbit haemorrhagic disease: field epidemiology and the management of wild rabbit populations. Rev Sci Tech 21: 347-358.

23. Forrester NL, Boag B, Moss SR, Turner SL, Trout RC, et al. (2003) Long-term survival of New Zealand rabbit haemorrhagic disease virus RNA in wild rabbits, revealed by RT-PCR and phylogenetic analysis. Journal of General Virology 84: 3079-3086.

24. Kerr PJ, Kitchen A, Holmes EC (2009) Origin and phylodynamics of rabbit hemorrhagic disease virus. Journal of Virology 83: 12129-12138.

25. Hicks AL, Duffy S (2012) One misdated sequence of rabbit hemorrhagic disease virus prevents accurate estimation of its nucleotide substitution rate. Bmc Evolutionary Biology 12: 74.

26. Navas-Martin SR, Weiss S (2004) Coronavirus replication and pathogenesis: Implications for the recent outbreak of severe acute respiratory syndrome (SARS), and the challenge for vaccine development. J Neurovirol 10: 75-85.

27. Weiss SR, Navas-Martin S (2005) Coronavirus pathogenesis and the emerging pathogen severe acute respiratory syndrome coronavirus. Microbiol Mol Biol Rev 69: 635-664.

28. Lai MMC, Perlman S, Anderson LJ (2007) *Coronaviridae*. In: Knipe DM, Howley PM, Griffin DE, Lamb RA, Martin MA et al., editors. Fields Virology. 5th ed. Philadelphia, PA: Lippincott Williams & Wilkins. pp. 1305-1335.

29. Park SJ, Kim GY, Choy HE, Hong YJ, Saif LJ, et al. (2007) Dual enteric and respiratory tropisms of winter dysentery bovine coronavirus in calves. Archives of Virology 152: 1885-1900.

30. Tsunemitsu H, Yonemichi H, Hirai T, Kudo T, Onoe S, et al. (1991) Isolation of bovine coronavirus from feces and nasal swabs of calves with diarrhea. J Vet Med Sci 53: 433-437.

31. Rekik MR, Dea S (1994) Comparative sequence analysis of a polymorphic region of the spike glycoprotein S1 subunit of enteric bovine coronavirus isolates. Archives of Virology 135: 319-331.

32. Gubler DJ, Kuno G, Markoff L (2007) Flaviviruses. In: Knipe DM, Howley PM, Griffin DE, Lamb RA, Martin MA et al., editors. Fields Virology. 5th ed. Philadelphia, PA: Lippincott Williams & Wilkins. pp. 1153-1252.

33. Martina BE, Koraka P, Osterhaus AD (2009) Dengue virus pathogenesis: an integrated view. Clin Microbiol Rev 22: 564-581.

34. Gould EA, Solomon T (2008) Pathogenic flaviviruses. Lancet 371: 500-509.

35. Costa RL, Voloch CM, Schrago CG (2012) Comparative evolutionary epidemiology of dengue virus serotypes. Infect Genet Evol 12: 309-314.

36. Sall AA, Faye O, Diallo M, Firth C, Kitchen A, et al. (2010) Yellow Fever Virus Exhibits Slower Evolutionary Dynamics than Dengue Virus. Journal of Virology 84: 765-772.

37. Allicock OM, Lemey P, Tatem AJ, Pybus OG, Bennett SN, et al. (2012) Phylogeography and population dynamics of dengue viruses in the Americas. Molecular Biology and Evolution 29: 1533-1543.

38. Chen SP (2012) Molecular evolution and epidemiology of four serotypes of dengue virus in Thailand from 1973 to 2007. Epidemiol Infect: 1-6.

39. Anez G, Morales-Betoulle ME, Rios M (2011) Circulation of different lineages of dengue virus type 2 in Central America, their evolutionary time-scale and selection pressure analysis. Plos One 6: e27459.

40. Kumar SR, Patil JA, Cecilia D, Cherian SS, Barde PV, et al. (2010) Evolution, dispersal and replacement of American genotype dengue type 2 viruses in India (1956-2005): selection pressure and molecular clock analyses. Journal of General Virology 91: 707-720.

41. Zhang C, Mammen MP, Jr., Chinnawirotpisan P, Klungthong C, Rodpradit P, et al. (2006) Structure and age of genetic diversity of dengue virus type 2 in Thailand. Journal of General Virology 87: 873-883.

42. Carrington CV, Foster JE, Pybus OG, Bennett SN, Holmes EC (2005) Invasion and maintenance of dengue virus type 2 and type 4 in the Americas. Journal of Virology 79: 14680-14687.

43. Patil JA, Cherian S, Walimbe AM, Bhagat A, Vallentyne J, et al. (2012) Influence of evolutionary events on the Indian subcontinent on the phylogeography of dengue type 3 and 4 viruses. Infect Genet Evol 12: 1759-1769.

44. de Araujo JM, Bello G, Romero H, Nogueira RM (2012) Origin and evolution of dengue virus type 3 in Brazil. PLoS Negl Trop Dis 6: e1784.

45. Araujo JMG, Nogueira RMR, Schatzmayr HG, Zanotto PMD, Bello G (2009) Phylogeography and evolutionary history of dengue virus type 3. Infection Genetics and Evolution 9: 716-725.

46. Solomon T, Vaughn DW (2002) Pathogenesis and clinical features of Japanese encephalitis and West Nile virus infections. Curr Top Microbiol Immunol 267: 171-194.

47. Johnson RT (1987) The pathogenesis of acute viral encephalitis and postinfectious encephalomyelitis. J Infect Dis 155: 359-364.

48. Chen SP (2012) Molecular phylogenetic and evolutionary analysis of Japanese encephalitis virus in China. Epidemiol Infect 140: 1637-1643.

49. Mansfield KL, Johnson N, Phipps LP, Stephenson JR, Fooks AR, et al. (2009) Tick-borne encephalitis virus - a review of an emerging zoonosis. Journal of General Virology 90: 1781-1794.

50. Subbotina EL, Loktev VB (2012) Molecular Evolution of the Tick-Borne Encephalitis and Powassan Viruses. Molecular Biology 46: 75-84.

51. Weidmann M, Ruzek D, Krivanec K, Zoller G, Essbauer S, et al. (2011) Relation of genetic phylogeny and geographical distance of tick-borne encephalitis virus in central Europe. Journal of General Virology 92: 1906-1916.

52. Uzcategui NY, Sironen T, Golovljova I, Jaaskelainen AE, Valimaa H, et al. (2012) Rate of evolution and molecular epidemiology of tick-borne encephalitis virus in Europe, including two isolations from the same focus 44 years apart. Journal of General Virology 93: 786-796.

53. Ebel GD (2010) Update on Powassan virus: emergence of a North American tick-borne flavivirus. Annu Rev Entomol 55: 95-110.

54. Monath TP, Barrett AD (2003) Pathogenesis and pathophysiology of yellow fever. Advances in Virus Research, Vol 57 60: 343-395.

55. Lemon SM, Walker C, Alter M, Yi M (2007) Hepatitis C virus. In: Knipe DM, Howley PM, Griffin DE, Lamb RA, Martin MA et al., editors. Fields Virology. 5th ed. Philadelphia, PA: Lippincott Williams & Wilkins. pp. 1253-1304.

56. Gray RR, Parker J, Lemey P, Salemi M, Katzourakis A, et al. (2011) The mode and tempo of hepatitis C virus evolution within and among hosts. Bmc Evolutionary Biology 11.

57. Culasso AC, Elizalde M, Campos RH, Barbini L (2012) Molecular survey of hepatitis C virus in the touristic city of Mar del Plata, Argentina. Plos One 7: e44757.

58. Emerson SU, Purcell RH (2007) Hepatitis E virus. In: Knipe DM, Howley PM, Griffin DE, Lamb RA, Martin MA et al., editors. Fields Virology. 5th ed. Philadelphia, PA: Lippincott Williams & Wilkins. pp. 3048-3058.

59. Chauhan A, Jameel S, Dilawari JB, Chawla YK, Kaur U, et al. (1993) Hepatitis E virus transmission to a volunteer. Lancet 341: 149-150.

60. Purdy MA, Khudyakov YE (2010) Evolutionary History and Population Dynamics of Hepatitis E Virus. Plos One 5.

61. Mason PW, Grubman MJ, Baxt B (2003) Molecular basis of pathogenesis of FMDV. Virus Res 91: 9-32.

62. Saiz M, Nunez JI, Jimenez-Clavero MA, Baranowski E, Sobrino F (2002) Foot-and-mouth disease virus: biology and prospects for disease control. Microbes Infect 4: 1183-1192.

63. Whitton JL, Cornell CT, Feuer R (2005) Host and virus determinants of picornavirus pathogenesis and tropism. Nat Rev Microbiol 3: 765-776.

64. Tully DC, Fares MA (2008) The tale of a modern animal plague: Tracing the evolutionary history and determining the time-scale for foot and mouth disease virus. Virology 382: 250-256.

65. Yoon SH, Lee KN, Park JH, Kim H (2011) Molecular epidemiology of foot-and-mouth disease virus serotypes A and O with emphasis on Korean isolates: temporal and spatial dynamics. Archives of Virology 156: 817-826.

66. Hicks AL, Duffy S (2011) Genus-specific substitution rate variability among picornaviruses. Journal of Virology 85: 7942-7947.

67. Sangula AK, Belsham GJ, Muwanika VB, Heller R, Balinda SN, et al. (2010) Co-circulation of two extremely divergent serotype SAT 2 lineages in Kenya highlights challenges to foot-and-mouth disease control. Archives of Virology 155: 1625-1630.

68. Pallansch M, Roos R (2007) Enteroviruses, polioviruses, coxsackieviruses, echoviruses, and newer enteroviruses. In: Knipe DM, Howley PM, Griffin DE, Lamb RA, Martin MA et al., editors. Fields Virology. 5th ed. Philadelphia, PA: Lippincott Williams & Wilkins. pp. 839-893.

69. Brown EH (1973) Enterovirus infections. Br Med J 2: 169-171.

70. Racaniello VR (1995) Early events in infection: Receptor binding and cell entry. In: Rotbart HA, editor. Human Enterovirus Infections. Washington, DC: ASM Press. pp. 73-93.

71. Morens D, Pallanasch M (1995) Epidemiology. In: Rotbart HA, editor. Human Enterovirus Infections. Washington, DC: ASM Press. pp. 2-23.

72. Smura T, Ylipaasto P, Klemola P, Kaijalainen S, Kyllonen L, et al. (2010) Cellular tropism of human enterovirus D species serotypes EV-94, EV-70, and EV-68 in vitro: implications for pathogenesis. J Med Virol 82: 1940-1949.

73. Jacques J, Moret H, Minette D, Leveque N, Jovenin N, et al. (2008) Epidemiological, molecular, and clinical features of enterovirus respiratory infections in French children between 1999 and 2005. Journal of Clinical Microbiology 46: 206-213.

74. Tee KK, Lam TTY, Chan YF, Bible JM, Kamarulzaman A, et al. (2010) Evolutionary Genetics of Human Enterovirus 71: Origin, Population Dynamics, Natural Selection, and Seasonal Periodicity of the VP1 Gene. Journal of Virology 84: 3339-3350.

75. Mirand A, Schuffenecker I, Henquell C, Billaud G, Jugie G, et al. (2010) Phylogenetic evidence for a recent spread of two populations of human enterovirus 71 in European countries. Journal of General Virology 91: 2263-2277.

76. Pinkert S, Klingel K, Lindig V, Dorner A, Zeichhardt H, et al. (2011) Virus-host coevolution in a persistently coxsackievirus B3-infected cardiomyocyte cell line. Journal of Virology 85: 13409-13419.

77. Klingel K, Kandolf R (1993) The role of enterovirus replication in the development of acute and chronic heart muscle disease in different immunocompetent mouse strains. Scand J Infect Dis Suppl 88: 79-85.

78. Kandolf R, Klingel K, Zell R, Selinka HC, Raab U, et al. (1993) Molecular pathogenesis of enterovirus-induced myocarditis: virus persistence and chronic inflammation. Intervirology 35: 140-151.

79. Chapman NM, Kim KS (2008) Persistent coxsackievirus infection: enterovirus persistence in chronic myocarditis and dilated cardiomyopathy. Curr Top Microbiol Immunol 323: 275-292.

80. Chu PY, Ke GM, Chen YS, Lu PL, Chen HL, et al. (2010) Molecular epidemiology of Coxsackievirus B3. Infection Genetics and Evolution 10: 777-784.

81. Gullberg M, Tolf C, Jonsson N, Mulders MN, Savolainen-Kopra C, et al. (2010) Characterization of a Putative Ancestor of Coxsackievirus B5. Journal of Virology 84: 9695-9708.

82. Leitch ECM, Cabrerizo M, Cardosa J, Harvala H, Ivanova OE, et al. (2010) Evolutionary Dynamics and Temporal/Geographical Correlates of Recombination in the Human Enterovirus Echovirus Types 9, 11, and 30. Journal of Virology 84: 9292-9300.

83. Knowles NJ, Hovi T, Hyypia T, King AM, Lindberg AM, et al. (2012) *Picornaviridae*. In: King AM, Adams MJ, Carstens EB, Lefkowitz EJ, editors. Virus Taxonomy: Ninth Report of the International Committee on Taxonomy of Viruses. Oxford, UK: Elsevier. pp. 855-880.

84. Escribano-Romero E, Jimenez-Clavero MA, Ley V (2000) Swine vesicular disease virus. Pathology of the disease and molecular characteristics of the virion. Anim Health Res Rev 1: 119-126.

85. Lin F, Mackay DK, Knowles NJ (1998) The persistence of swine vesicular disease virus infection in pigs. Epidemiol Infect 121: 459-472.

86. Tokarz R, Firth C, Madhi SA, Howie SR, Wu W, et al. (2012) Worldwide emergence of multiple clades of enterovirus 68. Journal of General Virology 93: 1952-1958.

87. Linsuwanon P, Puenpa J, Suwannakarn K, Auksornkitti V, Vichiwattana P, et al. (2012) Molecular epidemiology and evolution of human enterovirus serotype 68 in Thailand, 2006-2011. Plos One 7: e35190.

88. Hollinger F, Emerson SU (2007) Hepatitis A virus. In: Knipe DM, Howley PM, Griffin DE, Lamb RA, Martin MA et al., editors. Fields Virology. 5th ed. Philadelphia, PA: Lippincott Williams & Wilkins. pp. 911-947.

89. Harvala H, Robertson I, McWilliam Leitch EC, Benschop K, Wolthers KC, et al. (2008) Epidemiology and clinical associations of human parechovirus respiratory infections. Journal of Clinical Microbiology 46: 3446-3453.

90. Calvert J, Chieochansin T, Benschop KS, McWilliam Leitch EC, Drexler JF, et al. (2010) Recombination dynamics of human parechoviruses: investigation of type-specific differences in frequency and epidemiological correlates. Journal of General Virology 91: 1229-1238.

91. Faria NR, de Vries M, van Hemert FJ, Benschop K, van der Hoek L (2009) Rooting human parechovirus evolution in time. Bmc Evolutionary Biology 9.

92. Bangari DS, Pogranichniy RM, Gillespie T, Stevenson GW (2010) Genotyping of Porcine teschovirus from nervous tissue of pigs with and without polioencephalomyelitis in Indiana. J Vet Diagn Invest 22: 594-597.

93. Chiu SC, Hu SC, Chang CC, Chang CY, Huang CC, et al. (2012) The role of porcine teschovirus in causing diseases in endemically infected pigs. Veterinary Microbiology 161: 88-95.

94. Cano-Gomez C, Palero F, Buitrago MD, Garcia-Casado MA, Fernandez-Pinero J, et al. (2011) Analyzing the genetic diversity of teschoviruses in Spanish pig populations using complete VP1 sequences. Infection Genetics and Evolution 11: 2144-2150.

95. Wang B, Tian ZJ, Gong DQ, Li DY, Wang Y, et al. (2010) Isolation of serotype 2 porcine teschovirus in China: evidence of natural recombination. Veterinary Microbiology 146: 138-143.

96. Cano-Gomez C, Garcia-Casado MA, Soriguer R, Palero F, Jimenez-Clavero MA (2012) Teschoviruses and sapeloviruses in faecal samples from wild boar in Spain. Veterinary Microbiology.

97. Schwartz O, Albert ML (2010) Biology and pathogenesis of chikungunya virus. Nat Rev Microbiol 8: 491-500.

98. Ozden S, Huerre M, Riviere JP, Coffey LL, Afonso PV, et al. (2007) Human muscle satellite cells as targets of Chikungunya virus infection. Plos One 2: e527.

99. Griffin DE (2007) Alphaviruses. In: Knipe DM, Howley PM, Griffin DE, Lamb RA, Martin MA et al., editors. Fields Virology. 5th ed. Philadelphia, PA: Lippincott Williams & Wilkins. pp. 1023-1067.

100. Cherian SS, Walimbe AM, Jadhav SM, Gandhe SS, Hundekar SL, et al. (2009) Evolutionary rates and timescale comparison of Chikungunya viruses inferred from the whole genome/E1 gene with special reference to the 2005-07 outbreak in the Indian subcontinent. Infect Genet Evol 9: 16-23.

101. Russell RC (2002) Ross River virus: ecology and distribution. Annu Rev Entomol 47: 1-31.

102. Charles PC, Walters E, Margolis F, Johnston RE (1995) Mechanism of neuroinvasion of Venezuelan equine encephalitis virus in the mouse. Virology 208: 662-671.

103. Jackson AC, SenGupta SK, Smith JF (1991) Pathogenesis of Venezuelan equine encephalitis virus infection in mice and hamsters. Vet Pathol 28: 410-418.

104. Weaver SC, Ferro C, Barrera R, Boshell J, Navarro JC (2004) Venezuelan equine encephalitis. Annu Rev Entomol 49: 141-174.

105. Weaver SC, Rico-Hesse R, Scott TW (1992) Genetic diversity and slow rates of evolution in New World alphaviruses. Curr Top Microbiol Immunol 176: 99-117.

106. Weaver SC, Barrett ADT (2004) Transmission cycles, host range, evolution and emergence of arboviral disease. Nature Reviews Microbiology 2: 789-801.

107. Kubes V, Rios FA (1939) The Causative Agent of Infectious Equine Encephalomyelitis in Venezuela. Science 90: 20-21.

108. Liu C, Voth DW, Rodina P, Shauf LR, Gonzalez G (1970) A comparative study of the pathogenesis of western equine and eastern equine encephalomyelitis viral infections in mice by intracerebral and subcutaneous inoculations. J Infect Dis 122: 53-63.

109. Lockart RZ, Jr. (1963) Production of an Interferon by L Cells Infected with Western Equine Encephalomyelitis Virus. J Bacteriol 85: 556-566.

110. Meyer KF, Haring CM, Howitt B (1931) The Etiology of Epizootic Encephalomyelitis of Horses in the San Joaquin Valley, 1930. Science 74: 227-228.

111. Dent PB, Olson GB, Good RA, Rawls WE, South MA, et al. (1968) Rubella-virus/leucocyte interaction and its role in the pathogenesis of congenital syndrome. The Lancet 291: 291-293.

112. Chantler JK, Tingle AJ, Petty RE (1985) Persistent rubella virus infection associated with chronic arthritis in children. N Engl J Med 313: 1117-1123.

113. Chantler JK, da Roza DM, Bonnie ME, Reid GD, Ford DK (1985) Sequential studies on synovial lymphocyte stimulation by rubella antigen, and rubella virus isolation in an adult with persistent arthritis. Ann Rheum Dis 44: 564-568.

114. Nathanson N, Griffin DE (2007) Virus-induced immunosuppression. In: Nathanson N, Ahmed R, Biron CA, Brinton MA, Gonzalez-Scarano F et al., editors. Viral Pathogenesis and Immunity. 2nd ed. Oxford, UK: Elsevier. pp. 99-110.

115. Hobman T, Chantler J (2007) Rubella virus. In: Knipe DM, Howley PM, Griffin DE, Lamb RA, Martin MA et al., editors. Fields Virology. 5th ed. Philadelphia, PA: Lippincott Williams & Wilkins. pp. 1069-1100.

116. Buchmeier MJ, de la Torre JC, Peters CJ (2007) *Arenaviridae*: The viruses and their replication. In: Knipe DM, Howley PM, Griffin DE, Lamb RA, Martin MA et al., editors. Fields Virology. 5th ed. Philadelphia, PA: Lippincott Williams & Wilkins. pp. 1791-1827.

117. Russier M, Reynard S, Tordo N, Baize S (2012) NK cells are strongly activated by Lassa and Mopeia virus-infected human macrophages in vitro but do not mediate virus suppression. Eur J Immunol 42: 1822-1832.

118. Albarino CG, Palacios G, Khristova ML, Erickson BR, Carroll SA, et al. (2010) High diversity and ancient common ancestry of lymphocytic choriomeningitis virus. Emerg Infect Dis 16: 1093-1100.

119. Lipkin WI, Briese T (2007) *Bornaviridae*. In: Knipe DM, Howley PM, Griffin DE, Lamb RA, Martin MA et al., editors. Fields Virology. 5th ed. Philadelphia, PA: Lippincott Williams & Wilkins. pp. 1829-1851.

120. Lipkin WI, Briese T, Hornig M (2011) Borna disease virus - fact and fantasy. Virus Res 162: 162-172.

121. Staeheli P, Sauder C, Hausmann J, Ehrensperger F, Schwemmle M (2000) Epidemiology of Borna disease virus. Journal of General Virology 81: 2123-2135.

122. Rackova S, Janu L, Kabickova H (2010) Borna disease virus (BDV) circulating immunocomplex positivity in addicted patients in the Czech Republic: a prospective cohort analysis. BMC Psychiatry 10: 70.

123. Richt JA, Pfeuffer I, Christ M, Frese K, Bechter K, et al. (1997) Borna disease virus infection in animals and humans. Emerg Infect Dis 3: 343-352.

124. Valbuena G, Walker DH (2006) The endothelium as a target for infections. Annu Rev Pathol 1: 171-198.

125. Schmaljohn CS, Nichol ST (2007) *Bunyaviridae*. In: Knipe DM, Howley PM, Griffin DE, Lamb RA, Martin MA et al., editors. Fields Virology. 5th ed. Philadelphia, PA: Lippincott Williams & Wilkins. pp. 1741-1789.

126. Klingstrom J, Heyman P, Escutenaire S, Sjolander KB, De Jaegere F, et al. (2002) Rodent host specificity of European hantaviruses: evidence of Puumala virus interspecific spillover. J Med Virol 68: 581-588.

127. Ramsden C, Melo FL, Figueiredo LM, Holmes EC, Zanotto PM (2008) High rates of molecular evolution in hantaviruses. Molecular Biology and Evolution 25: 1488-1492.

128. Connolly-Andersen AM, Douagi I, Kraus AA, Mirazimi A (2009) Crimean Congo hemorrhagic fever virus infects human monocyte-derived dendritic cells. Virology 390: 157-162.

129. Carroll SA, Bird BH, Rollin PE, Nichol ST (2010) Ancient common ancestry of Crimean-Congo hemorrhagic fever virus. Molecular Phylogenetics and Evolution 55: 1103-1110.

130. Pepin M, Bouloy M, Bird BH, Kemp A, Paweska J (2010) Rift Valley fever virus(Bunyaviridae: Phlebovirus): an update on pathogenesis, molecular epidemiology, vectors, diagnostics and prevention. Vet Res 41: 61.

131. Gommet C, Billecocq A, Jouvion G, Hasan M, Zaverucha do Valle T, et al. (2011) Tissue tropism and target cells of NSs-deleted rift valley fever virus in live immunodeficient mice. PLoS Negl Trop Dis 5: e1421.

132. Bird BH, Khristova ML, Rollin PE, Ksiazek TG, Nichol ST (2007) Complete genome analysis of 33 ecologically and biologically diverse Rift Valley fever virus strains reveals widespread virus movement and low genetic diversity due to recent common ancestry. Journal of Virology 81: 2805-2816.

133. Bird BH, Githinji JW, Macharia JM, Kasiiti JL, Muriithi RM, et al. (2008) Multiple virus lineages sharing recent common ancestry were associated with a Large Rift Valley fever outbreak among livestock in Kenya during 2006-2007. Journal of Virology 82: 11152-11166.

134. Collao X, Palacios G, Sanbonmatsu-Gamez S, Perez-Ruiz M, Negredo AI, et al. (2009) Genetic diversity of Toscana virus. Emerg Infect Dis 15: 574-577.

135. Zehender G, Bernini F, Delogu M, Cusi MG, Rezza G, et al. (2009) Bayesian skyline plot inference of the Toscana virus epidemic: a decline in the effective number of infections over the last 30 years. Infect Genet Evol 9: 562-566.

136. Palese P, Shaw ML (2007) *Orthomyxoviridae*: The viruses and their replication. In: Knipe DM, Howley PM, Griffin DE, Lamb RA, Martin MA et al., editors. Fields Virology. 5th ed. Philadelphia, PA: Lippincott Williams & Wilkins. pp. 1647-1689.

137. Wright PF, Neumann G, Kawaoka Y (2007) Orthomyxoviruses. In: Knipe DM, Howley PM, Griffin DE, Lamb RA, Martin MA et al., editors. Fields Virology. 5th ed. Philadelphia, PA: Lippincott Williams & Wilkins. pp. 1691-1740.

138. Chen RB, Holmes EC (2006) Avian influenza virus exhibits rapid evolutionary dynamics. Molecular Biology and Evolution 23: 2336-2341.

139. Chen R, Holmes EC (2010) Hitchhiking and the population genetic structure of avian influenza virus. J Mol Evol 70: 98-105.

140. Bahl J, Vijaykrishna D, Holmes EC, Smith GJ, Guan Y (2009) Gene flow and competitive exclusion of avian influenza A virus in natural reservoir hosts. Virology 390: 289-297.

141. Smith GJ, Vijaykrishna D, Bahl J, Lycett SJ, Worobey M, et al. (2009) Origins and evolutionary genomics of the 2009 swine-origin H1N1 influenza A epidemic. Nature 459: 1122-1125.

142. Vijaykrishna D, Bahl J, Riley S, Duan L, Zhang JX, et al. (2008) Evolutionary dynamics and emergence of panzootic H5N1 influenza viruses. Plos Pathogens 4: e1000161.

143. Chen RB, Holmes EC (2008) The evolutionary dynamics of human influenza B virus. Journal of Molecular Evolution 66: 655-663.

144. Gatherer D (2010) Tempo and mode in the molecular evolution of influenza C. PLoS Curr 2: RRN1199.

145. Beineke A, Puff C, Seehusen F, Baumgartner W (2009) Pathogenesis and immunopathology of systemic and nervous canine distemper. Vet Immunol Immunopathol 127: 1-18.

146. Griffin DE (2007) Measles virus. In: Knipe DM, Howley PM, Griffin DE, Lamb RA, Martin MA et al., editors. Fields Virology. 5th ed. Philadelphia, PA: Lippincott Williams & Wilkins. pp. 1551-1585.

147. Harder TC, Kenter M, Vos H, Siebelink K, Huisman W, et al. (1996) Canine distemper virus from diseased large felids: biological properties and phylogenetic relationships. Journal of General Virology 77 ( Pt 3): 397-405.

148. Monne I, Fusaro A, Valastro V, Citterio C, Pozza MD, et al. (2011) A distinct CDV genotype causing a major epidemic in Alpine wildlife. Veterinary Microbiology 150: 63-69.

149. Rima BK, Duprex WP (2006) Morbilliviruses and human disease. J Pathol 208: 199-214.

150. Pomeroy LW, Bjornstad ON, Holmes EC (2008) The evolutionary and epidemiological dynamics of the paramyxoviridae. Journal of Molecular Evolution 66: 98-106.

151. Saitoh M, Takeda M, Gotoh K, Takeuchi F, Sekizuka T, et al. (2012) Molecular evolution of hemagglutinin (H) gene in measles virus genotypes D3, D5, D9, and H1. Plos One 7: e50660.

152. Karron R, Collins P (2007) Parainfluenza viruses. In: Knipe DM, Howley PM, Griffin DE, Lamb RA, Martin MA et al., editors. Fields Virology. 5th ed. Philadelphia, PA: Lippincott Williams & Wilkins. pp. 1497-1526.

153. Beck ET, He J, Nelson MI, Bose ME, Fan J, et al. (2012) Genome sequencing and phylogenetic analysis of 39 human parainfluenza virus type 1 strains isolated from 1997-2010. Plos One 7: e46048.

154. Hviid A, Rubin S, Muhlemann K (2008) Mumps. Lancet 371: 932-944.

155. Carbone K, Rubin S (2007) Mumps virus. In: Knipe DM, Howley PM, Griffin DE, Lamb RA, Martin MA et al., editors. Fields Virology. 5th ed. Philadelphia, PA: Lippincott Williams & Wilkins. pp. 1527-1550.

156. Papenburg J, Boivin G (2010) The distinguishing features of human metapneumovirus and respiratory syncytial virus. Rev Med Virol 20: 245-260.

157. Collins PL, Crowe Jr. JE (2007) Respiratory syncytial virus and Metapneumovirus. In: Knipe DM, Howley PM, Griffin DE, Lamb RA, Martin MA et al., editors. Fields Virology. 5th ed. Philadelphia, PA: Lippincott Williams & Wilkins. pp. 1601-1646.

158. Li J, Ren L, Guo L, Xiang Z, Paranhos-Baccala G, et al. (2012) Evolutionary dynamics analysis of human metapneumovirus subtype A2: genetic evidence for its dominant epidemic. Plos One 7: e34544.

159. de Graaf M, Osterhaus ADME, Fouchier RAM, Holmes EC (2008) Evolutionary dynamics of human and avian metapneumoviruses. Journal of General Virology 89: 2933-2942.

160. Padhi A, Verghese B (2008) Positive natural selection in the evolution of human metapneumovirus attachment glycoprotein. Virus Res 131: 121-131.

161. Easton AJ, Domachowske JB, Rosenberg HF (2004) Animal pneumoviruses: molecular genetics and pathogenesis. Clin Microbiol Rev 17: 390-412.

162. Tan L, Lemey P, Houspie L, Viveen MC, Jansen NJG, et al. (2012) Genetic Variability among Complete Human Respiratory Syncytial Virus Subgroup A Genomes: Bridging Molecular Evolutionary Dynamics and Epidemiology. Plos One 7.

163. van der Westhuizen B (1967) Studies on bovine ephemeral fever. I. Isolation and preliminary characterization of a virus from natural and experimentally produced cases of bovine ephemeral fever. Onderstepoort J Vet Res 34: 29-40.

164. St George TD (1988) Bovine ephemeral fever: a review. Trop Anim Health Prod 20: 194-202.

165. Nandi S, Negi BS (1999) Bovine ephemeral fever: a review. Comp Immunol Microbiol Infect Dis 22: 81-91.

166. Lyles D, Rupprecht C (2007) *Rhabdoviridae*. In: Knipe DM, Howley PM, Griffin DE, Lamb RA, Martin MA et al., editors. Fields Virology. 5th ed. Philadelphia, PA: Lippincott Williams & Wilkins. pp. 1363-1408.

167. Bourhy H, Reynes JM, Dunham EJ, Dacheux L, Larrous F, et al. (2008) The origin and phylogeography of dog rabies virus. Journal of General Virology 89: 2673-2681.

168. Ming P, Yan J, Rayner S, Meng S, Xu G, et al. (2010) A history estimate and evolutionary analysis of rabies virus variants in China. Journal of General Virology 91: 759-764.

169. Talbi C, Holmes EC, de Benedictis P, Faye O, Nakoune E, et al. (2009) Evolutionary history and dynamics of dog rabies virus in western and central Africa. Journal of General Virology 90: 783-791.

170. Gong W, Jiang Y, Za Y, Zeng Z, Shao M, et al. (2010) Temporal and spatial dynamics of rabies viruses in China and Southeast Asia. Virus Res 150: 111-118.

171. Kobayashi Y, Suzuki Y, Itou T, Ito FH, Sakai T, et al. (2011) Evolutionary history of dog rabies in Brazil. Journal of General Virology 92: 85-90.

172. Fooks AR, Brookes SM, Johnson N, McElhinney LM, Hutson AM (2003) European bat lyssaviruses: an emerging zoonosis. Epidemiol Infect 131: 1029-1039.

173. Fooks AR, McElhinney LM, Pounder DJ, Finnegan CJ, Mansfield K, et al. (2003) Case report: isolation of a European bat lyssavirus type 2a from a fatal human case of rabies encephalitis. J Med Virol 71: 281-289.

174. Amengual B, Bourhy H, Lopez-Roig M, Serra-Cobo J (2007) Temporal dynamics of European bat Lyssavirus type 1 and survival of Myotis myotis bats in natural colonies. Plos One 2: e566.

175. Davis PL, Holmes EC, Larrous F, Van der Poel WH, Tjornehoj K, et al. (2005) Phylogeography, population dynamics, and molecular evolution of European bat lyssaviruses. Journal of Virology 79: 10487-10497.

176. Schwartz-Cornil I, Mertens PP, Contreras V, Hemati B, Pascale F, et al. (2008) Bluetongue virus: virology, pathogenesis and immunity. Vet Res 39: 46.

177. Roy P (2007) Orbiviruses. In: Knipe DM, Howley PM, Griffin DE, Lamb RA, Martin MA et al., editors. Fields Virology. 5th ed. Philadelphia, PA: Lippincott Williams & Wilkins. pp. 1975-1997.

178. Carpi G, Holmes EC, Kitchen A (2010) The evolutionary dynamics of bluetongue virus. J Mol Evol 70: 583-592.

179. Tsai K, Karstad L (1973) The pathogenesis of epizootic hemorrhagic disease of deer: an electron microscopic study. Am J Pathol 70: 379-400.

180. Fletch AL, Karstad LH (1971) Studies on the pathogenesis of experimental epizootic hemorrhagic disease of white-tailed deer. Can J Comp Med 35: 224-229.

181. Biek R (2007) Evolutionary dynamics and spatial genetic structure of epizootic hemorrhagic disease virus in the eastern United States. Infect Genet Evol 7: 651-655.

182. Estes MK, Kapikian AZ (2007) Rotaviruses. In: Knipe DM, Howley PM, Griffin DE, Lamb RA, Martin MA et al., editors. Fields Virology. 5th ed. Philadelphia, PA: Lippincott Williams & Wilkins. pp. 1917-1974.

183. Nagaoka Y, Tatsumi M, Tsugawa T, Yoto Y, Tsutsumi H (2012) Phylogenetic and computational structural analysis of VP7 gene of group a human rotavirus G1P[8] strains obtained in Sapporo, Japan from 1987 to 2000. J Med Virol 84: 832-838.

184. Matthijnssens J, Heylen E, Zeller M, Rahman M, Lemey P, et al. (2010) Phylodynamic Analyses of Rotavirus Genotypes G9 and G12 Underscore Their Potential for Swift Global Spread. Molecular Biology and Evolution 27: 2431-2436.

185. Lahon A, Walimbe AM, Chitambar SD (2012) Full genome analysis of group B rotaviruses from western India: Genetic relatedness and evolution. Journal of General Virology.
