# Supplementary material for: Cell Tropism Predicts Long-term Nucleotide Substitution Rates of Mammalian RNA Viruses
Source: PLoS Pathog. 2014 Jan 9;10(1):e1003838. doi: 10.1371/journal.ppat.1003838 (PMC3887100; doi:10.1371/journal.ppat.1003838)
Supplement: Table S2 — Dataset and analysis information for novel substitution rates produced in this study. Abbreviations for viruses and genes are as in Table S1. Nucleotide substitution models shown general time reversible (GTR), Tamura-Nei (TrN), transition (TIM), transversion (TVM), transversion with equal frequencies (TVMef), Kimura 3-parameter with unequal frequencies (K81uf), and Hasegawa-Kishino-Yano (HKY); corrections for invariant sites (+i) and a gamma distribution of rate heterogeneity (+G) were also included in some models. (DOCX) [file ppat.1003838.s005.docx]

**Table S2.** Dataset and analysis information for novel substitution rates produced in this study. Abbreviations for viruses and genes are as in Table S1. Nucleotide substitution models shown general time reversible (GTR), Tamura-Nei (TrN), transition (TIM), transversion (TVM), transversion with equal frequencies (TVMef), Kimura 3-parameter with unequal frequencies (K81uf), and Hasegawa-Kishino-Yano (HKY); corrections for invariant sites (+i) and a gamma distribution of rate heterogeneity (+G) were also included in some models.

| Virus | Gene | N_nucleotides_ | N_taxa_ | Date Range | Nucleotide Substitution Model | Clock Model | Demographic Model |
| --- | --- | --- | --- | --- | --- | --- | --- |
| hAstV | S (p) | 1071 | 86 | 1990-2010 | TrN+i+G | Uncorrelated lognormal | Bayesian skyline |
| EAV | S | 768 | 155 | 1954-2008 | TVM+i+G | Uncorrelated lognormal | Constant |
| PRRSV | NS | 1872 | 122 | 1990-2008 | GTR+i+G | Uncorrelated lognormal | Bayesian skyline |
| NoV GII.4 | NS | 1524 | 332 | 1971-2010 | GTR+i+G | Uncorrelated lognormal | Constant |
| bCoV | S (p) | 2703 | 45 | 1993-2011 | GTR+i+G | Uncorrelated lognormal | Exponential |
| JEV | NS | 2673 | 111 | 1935-2009 | GTR+i+G | Uncorrelated lognormal | Bayesian skyline |
| POWV | S | 660 | 66 | 1952-2011 | TIM+G | Uncorrelated lognormal | Bayesian skyline |
| TBEV | NS | 2709 | 65 | 1952-2010 | GTR+i+G | Uncorrelated lognormal | Bayesian skyline |
| YFV | NS | 2724 | 36 | 1973-2010 | GTR+i+G | Uncorrelated lognormal | Bayesian skyline |
| CVA16 | S | 891 | 394 | 1981-2010 | GTR+i+G | Uncorrelated lognormal | Bayesian skyline |
| CVA16 | NS (p) | 708 | 69 | 1997-2011 | GTR+i+G | Uncorrelated lognormal | Bayesian skyline |
| CVB4 | S | 807 | 100 | 1959-2010 | GTR+i+G | Uncorrelated lognormal | Bayesian skyline |
| E6 | S | 807 | 185 | 1991-2010 | GTR+G | Uncorrelated lognormal | Exponential |
| E9 | NS (p) | 549 | 83 | 1995-2010 | GTR+i+G | Uncorrelated lognormal | Bayesian skyline |
| E11 | NS (p) | 549 | 127 | 1982-2008 | GTR+i+G | Uncorrelated lognormal | Exponential |
| E13 | S | 807 | 64 | 1991-2006 | TrN+i+G | Uncorrelated lognormal | Constant |
| E30 | S | 810 | 421 | 1959-2010 | GTR+G | Uncorrelated lognormal | Bayesian skyline |
| E30 | NS (p) | 561 | 64 | 1981-2005 | GTR+i+G | Uncorrelated lognormal | Bayesian skyline |
| E33 | S | 807 | 44 | 1983-2005 | GTR+G | Uncorrelated lognormal | Exponential |
| SVDV | S | 897 | 51 | 1970-1999 | TrN+i+G | Uncorrelated lognormal | Bayesian skyline |
| CVA24 | S | 915 | 121 | 1963-2010 | GTR+i+G | Uncorrelated lognormal | Constant |
| CVA24 | NS | 549 | 236 | 1963-2010 | TrN+i+G | Uncorrelated lognormal | Constant |
| PV1 | S | 906 | 478 | 1959-2010 | GTR+G | Uncorrelated lognormal | Constant |
| PV1 | NS (p) | 792 | 35 | 1989-2008 | GTR+i+G | Uncorrelated lognormal | Constant |
| HAV | S | 900 | 213 | 1957-2010 | GTR+i+G | Uncorrelated lognormal | Exponential |
| HAV | NS | 1467 | 34 | 1957-2010 | GTR+i+G | Uncorrelated lognormal | Exponential |
| CHIKV | NS | 1833 | 151 | 1953-2011 | GTR+G | Uncorrelated lognormal | Exponential |
| RRV | S | 1266 | 137 | 1959-2009 | GTR+i | Uncorrelated lognormal | Constant |
| VEEV | S | 1269 | 40 | 1953-2008 | GTR+i+G | Uncorrelated lognormal | Constant |
| VEEV | NS | 1809 | 32 | 1943-2001 | GTR+i+G | Uncorrelated lognormal | Bayesian skyline |
| WEEV | S | 801 | 45 | 1930-2005 | GTR+G | Uncorrelated lognormal | Constant |
| RuV | S | 1416 | 194 | 1967-2007 | GTR+G | Uncorrelated lognormal | Bayesian skyline |
| LasV | S (p) | 771 | 50 | 1974-2009 | GTR+i+G | Uncorrelated lognormal | Exponential |
| BDV | S | 1110 | 44 | 1984-2008 | TVMef+i+G | Strict | Constant |
| CCHFV | NS | 11838 | 33 | 1956-2011 | GTR+i+G | Uncorrelated lognormal | Exponential |
| SEOV | S (p) | 1290 | 53 | 1995-2009 | TrN+i+G | Uncorrelated lognormal | Bayesian skyline |
| MeV | NS | 1524 | 94 | 1979-2009 | TIM+G | Uncorrelated lognormal | Bayesian skyline |
| MuV | S | 1746 | 51 | 1969-2009 | TVM+G | Uncorrelated lognormal | Bayesian skyline |
| BEFV | S | 1872 | 39 | 1966-2008 | GTR+G | Uncorrelated lognormal | Constant |
| RabV | NS (p) | 5979 | 35 | 1983-2009 | GTR+i+G | Uncorrelated lognormal | Bayesian skyline |
| RVA | NS | 3264 | 58 | 1974-2010 | GTR+i+G | Uncorrelated lognormal | Constant |
| RVA G2 | S | 972 | 117 | 1976-2007 | K81uf+G | Uncorrelated lognormal | Bayesian skyline |
| RVA G3 | S | 972 | 130 | 1974-2008 | HKY+G | Uncorrelated lognormal | Bayesian skyline |
| RVC | S | 1008 | 70 | 1986-2011 | GTR+i+G | Uncorrelated lognormal | Exponential |
| RVC | NS (p) | 450 | 46 | 1986-2009 | TIM+G | Uncorrelated lognormal | Constant |
