# Supplementary material for: Cell Tropism Predicts Long-term Nucleotide Substitution Rates of Mammalian RNA Viruses
Source: PLoS Pathog. 2014 Jan 9;10(1):e1003838. doi: 10.1371/journal.ppat.1003838 (PMC3887100; doi:10.1371/journal.ppat.1003838)
Supplement: Table S3 — Significant predictors of viral structural gene substitution rates using one rate per viral species. For each multiple regression analysis, the overall adjusted R 2 () of the model is given along with significant predictor variables (P<0.01) and their standardized coefficients (β) with 95% confidence intervals (CIs). In the first regression, the base levels were epithelial target cells, fecal-oral/respiratory transmission route, acute/persistent infection, species-specific host range, and dsRNA genome architecture. In the second regression, the base levels were neural target cells, bites/scratches transmission route, persistent infection, order-specific host range, and (−)ssRNA genome architecture. In the third regression, the base levels were leukocyte target cells, respiratory/vertical transmission route, acute infection, family-specific host range, and (+)ssRNA genome architecture. (DOCX) [file ppat.1003838.s006.docx]

**Table S3. Significant predictors of viral structural gene substitution rates** **using** **one rate per viral species**. For each multiple regression analysis, the overall adjusted *R*^2^ (
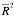
) of the model is given along with significant predictor variables (*P*<0.01) and their standardized coefficients (β) with 95% confidence intervals (CIs). In the first regression, the base levels were epithelial target cells, fecal-oral/respiratory transmission route, acute/persistent infection, species-specific host range, and dsRNA genome architecture. In the second regression, the base levels were neural target cells, bites/scratches transmission route, persistent infection, order-specific host range, and (-)ssRNA genome architecture. In the third regression, the base levels were leukocyte target cells, respiratory/vertical transmission route, acute infection, family-specific host range, and (+)ssRNA genome architecture.

|  | 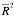 | Predictor | β (95% CI) | Significance |
| --- | --- | --- | --- | --- |
| 1 | 0.65 | Neurons | -0.76 (-1.14, -0.38) | <0.0001 |
|  |  |  |  |  |
|  |  |  |  |  |
| 2 | 0.65 | Epithelial cells | 1.01 (0.50, 1.51) | <0.0001 |
|  |  | Leukocytes | 0.51 (0.14, 0.89) | 0.009 |
| 3 | 0.65 | Neurons | -0.39 (-0.10, -0.68) | 0.009 |
|  |  | Epithelial cells | 0.49 (0.18, 0.69) | 0.009 |
