# Supplementary material for: Cell Tropism Predicts Long-term Nucleotide Substitution Rates of Mammalian RNA Viruses
Source: PLoS Pathog. 2014 Jan 9;10(1):e1003838. doi: 10.1371/journal.ppat.1003838 (PMC3887100; doi:10.1371/journal.ppat.1003838)
Supplement: Table S4 — Significant predictors of viral non-structural gene substitution rates using one rate per viral species. For each multiple regression analysis, the overall adjusted R 2 () of the model is given along with significant predictor variables (P<0.01) and their standardized coefficients (β) with 95% confidence intervals (CIs). In the first regression, the base levels were epithelial target cells, fecal-oral/respiratory transmission route, acute/persistent infection, species-specific host range, and dsRNA genome architecture. No factors were significant in this analysis. In the second regression, the base levels were neural target cells, bites/scratches transmission route, acute infection, order-specific host range, and (−)ssRNA genome architecture. No factors were significant in this analysis. In the third regression, the base levels were leukocyte target cells, respiratory/vertical transmission route, acute infection, family-specific host range, and (+)ssRNA genome architecture. (DOCX) [file ppat.1003838.s007.docx]

**Table S4.** **Significant predictors of viral non-structural gene substitution rates** **using** **one rate per viral species**. For each multiple regression analysis, the overall adjusted *R*^2^ (
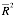
) of the model is given along with significant predictor variables (*P*<0.01) and their standardized coefficients (β) with 95% confidence intervals (CIs). In the first regression, the base levels were epithelial target cells, fecal-oral/respiratory transmission route, acute/persistent infection, species-specific host range, and dsRNA genome architecture. No factors were significant in this analysis. In the second regression, the base levels were neural target cells, bites/scratches transmission route, acute infection, order-specific host range, and (-)ssRNA genome architecture. No factors were significant in this analysis. In the third regression, the base levels were leukocyte target cells, respiratory/vertical transmission route, acute infection, family-specific host range, and (+)ssRNA genome architecture.

|  | 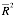 | Predictor | β (95% CI) | Significance |
| --- | --- | --- | --- | --- |
| 1 | 0.70 | - | - | - |
| 2 | 0.70 | - | - | - |
| 3 | 0.70 | Neurons | -0.47 (-0.75, -0.11) | 0.006 |
