# Supplementary material for: Cell Tropism Predicts Long-term Nucleotide Substitution Rates of Mammalian RNA Viruses
Source: PLoS Pathog. 2014 Jan 9;10(1):e1003838. doi: 10.1371/journal.ppat.1003838 (PMC3887100; doi:10.1371/journal.ppat.1003838)
Supplement: Table S5 — Significant predictors of viral substitution rates based on all rates included in this study. For each ANCOVA analysis, the overall adjusted R 2 () of the model is given along with the significant predictor variable (P<0.01) and its standardized coefficients (β) with 95% confidence intervals (CIs). In the first ANCOVA, the base levels were epithelial target cells, fecal-oral/respiratory transmission route, acute/persistent infection, species-specific host range, and dsRNA genome architecture. In the second ANCOVA, the base levels were neural target cells, bites/scratches transmission route, acute infection, order-specific host range, and (−)ssRNA genome architecture. In the third ANCOVA, the base levels were leukocyte target cells, respiratory/vertical transmission route, acute infection, family-specific host range, and (+)ssRNA genome architecture. (DOCX) [file ppat.1003838.s008.docx]

**Table S5. Significant predictors of viral substitution rates based on all rates included in this study**. For each ANCOVA analysis, the overall adjusted *R*^2^ (
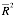
) of the model is given along with the significant predictor variable (*P*<0.01) and its standardized coefficients (β) with 95% confidence intervals (CIs). In the first ANCOVA, the base levels were epithelial target cells, fecal-oral/respiratory transmission route, acute/persistent infection, species-specific host range, and dsRNA genome architecture. In the second ANCOVA, the base levels were neural target cells, bites/scratches transmission route, acute infection, order-specific host range, and (-)ssRNA genome architecture. In the third ANCOVA, the base levels were leukocyte target cells, respiratory/vertical transmission route, acute infection, family-specific host range, and (+)ssRNA genome architecture.

|  | 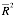 | Predictor | β (95% CI) | Significance |
| --- | --- | --- | --- | --- |
| 1 | 0.73 | Neurons | -0.71 (-0.90, -0.53) | <0.0001 |
|  |  | Leukocytes | -0.49 (-0.70, -0.28) | <0.0001 |
|  |  | Hepatocytes | -0.28 (-0.42, -0.13) | <0.0001 |
|  |  | Endothelial cells | -0.17 (-0.27, -0.06) | 0.002 |
| 2 | 0.73 | Epithelial cells | 1.05 (0.77, 1.32) | <0.0001 |
|  |  | Leukocytes | 0.51 (0.34, 0.69) | <0.0001 |
| 3 | 0.73 | Neurons | -0.37 (-0.49, -0.24) | <0.0001 |
|  |  | Epithelial cells | 0.51 (0.29, 0.73) | <0.0001 |
|  |  | Arthropod vector | -0.46 (-0.74, -0.17) | 0.002 |
