# Supplementary material for: Cell Tropism Predicts Long-term Nucleotide Substitution Rates of Mammalian RNA Viruses
Source: PLoS Pathog. 2014 Jan 9;10(1):e1003838. doi: 10.1371/journal.ppat.1003838 (PMC3887100; doi:10.1371/journal.ppat.1003838)
Supplement: Table S6 — Structural gene substitution rate variation among viruses with different cell tropisms. Based on the control datasets with one substitution rate per viral species. The significance of viruses with each target cell in the left column having higher log scale mean substitution rates than the viruses with each target cell in the top row is designated with a p-value from a one-tailed t-test. The threshold for statistical significance (P<0.01) was Bonferroni-corrected to account for multiple comparisons (P = 1×10^−3^). N = neurons, En = endothelial cells, L = leukocytes, H = hepatocytes, Ep = epithelial cells. (DOCX) [file ppat.1003838.s009.docx]

**Table S6. Structural gene substitution rate variation among viruses with different cell tropisms.** Based on the control datasets with one substitution rate per viral species. The significance of viruses with each target cell in the left column having higher log scale mean substitution rates than the viruses with each target cell in the top row is designated with a p*-*value from a one-tailed t*-*test. The threshold for statistical significance (*P*<0.01) was Bonferroni-corrected to account for multiple comparisons (*P=*1x10^-3^). N=neurons, En=endothelial cells, L=leukocytes, H=hepatocytes, Ep=epithelial cells.

|  | N | En | L | H | Ep |
| --- | --- | --- | --- | --- | --- |
| N | - | 0.98 | 1.00 | 1.00 | 1.00 |
| En | 0.02 | - | 0.98 | 0.98 | 1.00 |
| L | **0.0002** | 0.02 | - | 0.91 | 1.00 |
| H | 0.001 | 0.02 | 0.09 | - | 0.92 |
| Ep | **>0.0001** | **>0.0001** | **0.0001** | 0.08 | - |
